# Diagnostic Performance of Artificial Intelligence for the Detection and Segmentation of Periapical Lesions on Cone-Beam Computed Tomography: A Systematic Review and Meta-Analysis

**DOI:** 10.3390/diagnostics16213606

**Published:** 2026-11-01

**Authors:** Mohammed Howait, Mey A. Al-Habib

**Affiliations:** Department of Endodontics, Faculty of Dentistry, King Abdulaziz University, P.O. Box 80200, Jeddah 21589, Saudi Arabia; mhowait@kau.edu.sa

**Keywords:** artificial intelligence, deep learning, machine learning, cone-beam computed tomography, periapical lesion, apical periodontitis, diagnostic accuracy, image segmentation, endodontics, meta-analysis

## Abstract

**Background/Objectives**: Cone-beam computed tomography (CBCT) improves periapical lesion detection, but its interpretation is expertise-dependent. This systematic review evaluated artificial intelligence (AI) for detecting, classifying, and segmenting periapical lesions on CBCT. **Methods**: PubMed, the Cochrane Library, Scopus, Web of Science, and Google Scholar were searched to 12 December 2025 (PROSPERO: CRD420251184440). Studies comparing AI with an expert- or annotation-based reference standard on human CBCT scans were eligible. Risk of bias was assessed with the Quality Assessment of Diagnostic Accuracy Studies-2 (QUADAS-2) tool, and certainty of evidence with the Grading of Recommendations Assessment, Development and Evaluation (GRADE) approach. Sensitivity and specificity were pooled with a bivariate model where complete 2 × 2 data were available, and all metrics with univariate random-effects models. **Results**: Eighteen studies were included (detection or classification, 16; segmentation, 6); 15 had unclear risk of bias in at least one domain, and most were retrospective and internally validated. In the bivariate model (seven studies), sensitivity was 88.2% (95% confidence interval [CI]: 77.6–94.2%) and specificity 85.1% (73.4–92.1%). Univariate pooled sensitivity was 90.9% (84.2–94.9%; 14 studies), specificity 89.7% (83.2–93.9%; 11 studies), accuracy 86.7% (78.9–92.0%; 10 studies), and F1-score 83.7% (76.4–89.1%; seven studies). The pooled lesion Dice similarity coefficient was 0.70 (0.61–0.78; six studies), indicating moderate spatial overlap with reference annotations. Heterogeneity was considerable for detection metrics (I^2^ = 90–96%), prediction intervals were wide (sensitivity 48–99%), and the certainty of evidence was very low. **Conclusions**: AI shows promising performance for CBCT-based periapical lesion detection under research conditions, whereas segmentation overlap is moderate. Equivalence to clinicians and clinical effectiveness are not established; prospective, externally validated studies with standardized reference standards are needed.

## 1. Introduction

Periapical lesions are inflammatory conditions of the periradicular tissues, most commonly apical periodontitis, that develop in response to microbial infection of the root canal system [1,2,3]. Apical periodontitis is highly prevalent and is present in at least one tooth in approximately half of adults [4]. Accurate diagnosis underpins treatment planning, prognostic assessment, and the monitoring of healing [5], whereas undetected or inadequately managed lesions may lead to tooth loss, persistent pain, and possible systemic associations [6].

In this review, the term “periapical lesion” is used as an umbrella radiological term for periapical radiolucencies of inflammatory origin that are detectable on cone-beam computed tomography (CBCT); it encompasses apical periodontitis in its granulomatous and cystic forms and periapical abscesses. The clinical diagnosis “apical periodontitis” is used only where a primary study defined it as the target condition. The two terms are therefore related but not synonymous, and they are used consistently in this sense throughout.

CBCT provides three-dimensional (3D) visualization of dentoalveolar structures and eliminates the anatomical superimposition inherent to two-dimensional radiography [7]. It detects periapical bone changes more reliably than periapical or panoramic radiographs and improves the anatomical localization of lesions [8,9]. However, CBCT interpretation is time-consuming, requires specific training, and is subject to inter-observer variation and to difficulty in distinguishing subtle pathological changes from anatomical variants [10]. These limitations have stimulated interest in computer-assisted interpretation.

Artificial intelligence (AI), particularly deep learning (DL) based on convolutional neural networks (CNNs) and, more recently, transformer architectures, is now an established approach in medical image analysis [11,12]. In dentistry, AI has been applied to caries detection, periodontal bone-loss assessment, and the identification of oral pathology [13]. Applied to CBCT, AI models can process volumetric data to detect, classify, grade, or segment periapical lesions, with the potential to improve consistency and efficiency [14,15].

Studies of AI for periapical lesion assessment on CBCT have multiplied, but they differ in AI task, architecture, unit of analysis, reference standard, and validation strategy, and they report a variety of non-equivalent performance metrics. Previous reviews have pooled evidence across two- and three-dimensional imaging modalities [16] or synthesized it narratively [17]; none has synthesized CBCT evidence separately for detection and segmentation tasks while appraising risk of bias with a diagnostic-accuracy tool.

This systematic review and meta-analysis therefore aimed to evaluate the performance of AI models applied to CBCT for (1) the detection and classification of periapical lesions, expressed as sensitivity, specificity, accuracy, F1-score, and the area under the receiver operating characteristic curve (AUC); and (2) the segmentation of periapical lesions, expressed as the Dice similarity coefficient (DSC).

## 2. Materials and Methods

### 2.1. Protocol and Registration

**Figure 1:**
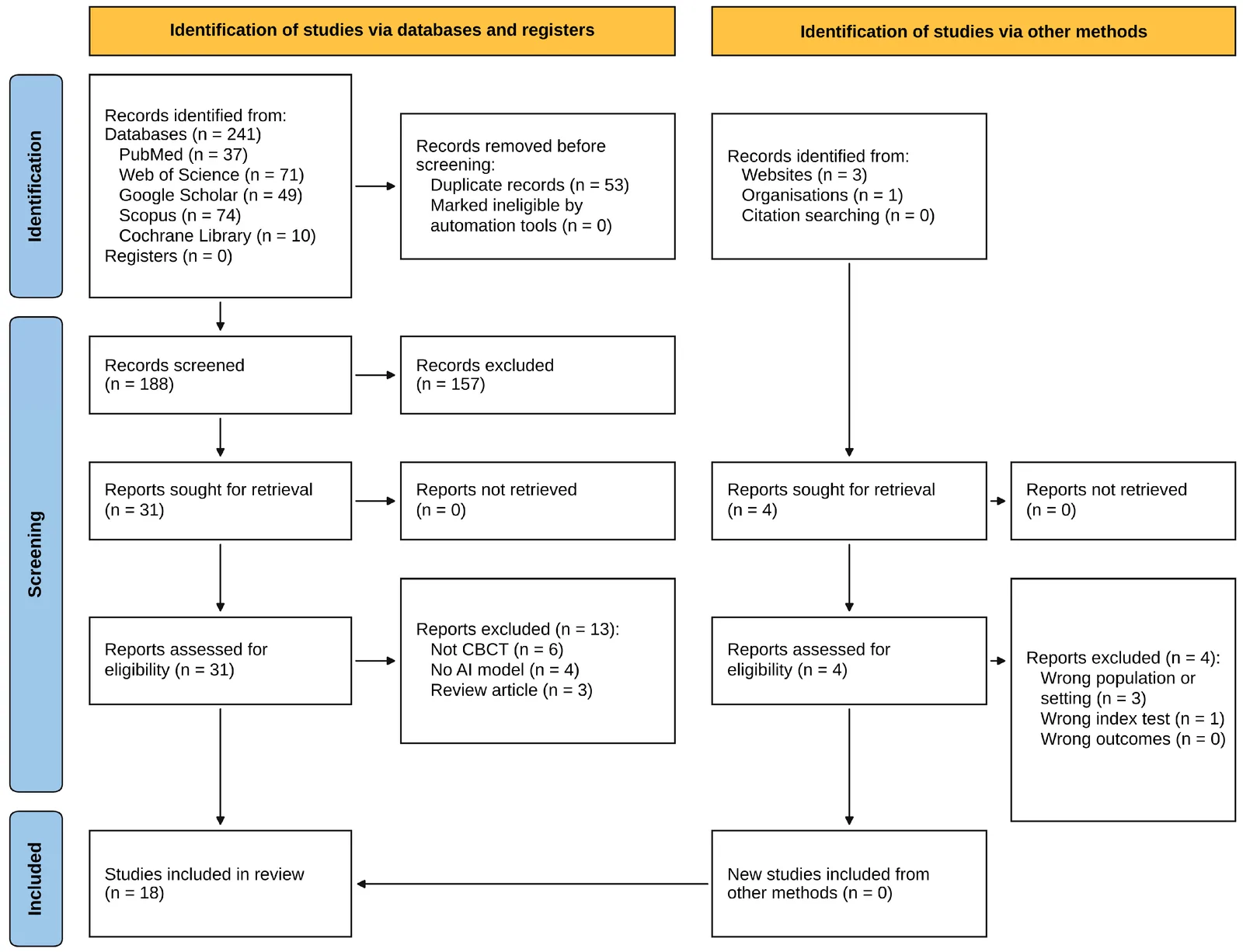
PRISMA 2020 flow diagram of study identification, screening, and inclusion. PRISMA, Preferred Reporting Items for Systematic Reviews and Meta-Analyses; AI, artificial intelligence; CBCT, cone-beam computed tomography.

This systematic review and meta-analysis was conducted and reported in accordance with the Preferred Reporting Items for Systematic Reviews and Meta-Analyses (PRISMA) 2020 statement [18] and its extension for diagnostic test accuracy studies (PRISMA-DTA) [19]. The completed PRISMA 2020 and PRISMA 2020 for Abstracts checklists and the PRISMA-DTA and PRISMA-DTA for Abstracts checklists are provided in the Supplementary Materials (Tables S8–S11), and the PRISMA 2020 flow diagram is shown in Figure 1. The protocol was registered in the International Prospective Register of Systematic Reviews (PROSPERO; CRD420251184440) in December 2025, and the protocol is publicly accessible at https://www.crd.york.ac.uk/PROSPERO/view/CRD420251184440 (accessed on 12 December 2025). Deviations from the registered protocol: risk of bias was assessed with the Quality Assessment of Diagnostic Accuracy Studies-2 (QUADAS-2) tool rather than the originally planned tool, detection/classification and segmentation outcomes were analyzed separately, and the certainty of evidence was assessed with the Grading of Recommendations Assessment, Development and Evaluation (GRADE) approach.

### 2.2. Eligibility Criteria

Eligibility was defined using the PIRD framework (Population, Index test, Reference standard, Diagnosis of interest) recommended for diagnostic test accuracy reviews [20]:

Population: CBCT scans of human teeth and jaws.

Index test: any AI model, including conventional machine learning (ML), DL (CNN-based, transfer-learning, and transformer-based architectures), and commercial AI platforms, applied to CBCT data to detect, classify, grade, localize, or segment periapical lesions.

Reference standard: expert clinical or radiological interpretation of CBCT, consensus annotation, manual or semi-automated lesion segmentation, or histopathology, as appropriate to the AI task.

Diagnosis of interest (target condition): periapical lesions, including apical periodontitis, periapical radiolucencies, granulomas, cysts, and abscesses.

Outcomes: for detection and classification: sensitivity (recall), specificity, accuracy, precision (positive predictive value, PPV), F1-score, and AUC; for segmentation: DSC and related overlap metrics.

Study design: diagnostic accuracy studies and AI model-development or validation studies, retrospective or prospective, reporting at least one quantitative performance metric.

Narrative and systematic reviews, editorials, case reports, conference abstracts without extractable data, and studies restricted to two-dimensional imaging were excluded. No language restriction was applied.

### 2.3. Information Sources and Search Strategy

PubMed, the Cochrane Library (Cochrane Central Register of Controlled Trials), Scopus, and Web of Science were searched from inception to 12 December 2025, the date on which all sources were last searched. The search combined three concept blocks (AI, CBCT, and periapical lesions) using free-text terms and Boolean operators; in PubMed, automatic term mapping to Medical Subject Headings was applied. The PubMed strategy was adapted to the syntax of each database; Web of Science was searched with the same three concept blocks adapted to its syntax. No date, language, or publication-type limits were applied, except that the Cochrane Library search was restricted to the Trials (CENTRAL) collection. Google Scholar was searched with the full string on the same date. Because Google Scholar does not support exhaustive retrieval, the protocol prespecified that the first 20 result pages (200 records) sorted by relevance would be screened; the search returned only 49 records in total, so all were screened and the prespecified cap was not reached. The complete strategies, with search dates and the number of records retrieved from each source, are provided in Table S1. ClinicalTrials.gov and the World Health Organization International Clinical Trials Registry Platform were searched for ongoing or unpublished studies, the websites of the American Association of Endodontists, the European Society of Endodontology, and the American Academy of Oral and Maxillofacial Radiology were searched for additional reports, and the reference lists of included studies were hand-searched.

### 2.4. Study Selection

Records were imported into Zotero (version 6.0.36; Corporation for Digital Scholarship, Vienna, VA, USA); duplicates were identified and merged manually, and retracted publications were excluded. Two reviewers (M.A.A.-H. and M.H.) independently screened titles and abstracts and then assessed full texts against the eligibility criteria; disagreements were resolved by discussion until consensus was reached. No automation tools were used. Reasons for full-text exclusion were recorded (Table S2).

### 2.5. Data Extraction

Data were extracted independently by two reviewers (M.H. and M.A.A.-H.) using a standardized, piloted form in Microsoft Excel 2021 (Microsoft Corp., Redmond, WA, USA). Extracted items were study identifier, design, country, sample size and characteristics, AI model and architecture (commercial or in-house), input data (two-dimensional slices or three-dimensional volumes), training-set size, AI task (detection, classification, grading, localization, or segmentation), reference standard (method, number and expertise of readers or annotators, use of consensus, calibration, and blinding to AI output), dataset partitioning, validation type (internal hold-out, cross-validation, independent test set, or external), unit of analysis (scan, image or slice, tooth, root, lesion, or voxel), and all reported performance metrics with their numerators and denominators. For every estimate, the denominator was the number of reference-standard-positive units (sensitivity), reference-standard-negative units (specificity), all test units (accuracy and F1-score), or test cases over which the metric was averaged (DSC); denominators were taken from the published counts or confusion matrices or, where only proportions and predictive values were reported, reconstructed from them. All values and denominators were verified against the full text of each article by both reviewers before analysis, and reconstructed or approximate denominators are identified in Table S7. No automation tools were used for data extraction.

When a study reported several models or configurations on the same test set, the configuration proposed by the authors as their final model was designated the primary estimate; architectures used only as benchmarks were not extracted. When tooth- and root-level results were both reported, the tooth level was designated primary because treatment decisions are made per tooth, and the root level was retained as a secondary estimate. When internal and external test sets were reported, the independent internal test set was designated as primary and external estimates were retained as secondary estimates. Only standalone AI performance was eligible for pooling; performance of clinicians assisted by AI was summarized narratively. Macro-averaged recall from multiclass grading tasks was not treated as binary sensitivity, voxel-level metrics from segmentation studies were not treated as lesion-level detection metrics, and detection rates in lesion-only samples were treated as sensitivity but not as accuracy. Where an ordinal grading output could be dichotomized into lesion present versus absent from a published confusion matrix, binary detection metrics were derived from it and identified as such.

### 2.6. Handling of Missing Data

The authors of the included studies were not contacted for missing or unclear data. Only data reported in the published articles and their Supplementary Materials were used; information that was not available was recorded as not reported (NR), and no missing values were imputed.

### 2.7. Risk of Bias and Applicability Assessment

Risk of bias was assessed independently by two reviewers with the QUADAS-2 tool [21], which covers four domains: patient selection, index test, reference standard, and flow and timing. Signaling questions were tailored to AI studies (Table 1). For the index test, reviewers considered whether performance was evaluated on data not used for model training or tuning and whether the operating threshold was prespecified; for the reference standard, they considered the number and expertise of readers or annotators, the use of consensus, and blinding to AI output. Each domain was judged as low, high, or unclear risk of bias; “unclear” was used when reporting was insufficient for a definitive judgement. The overall judgement for each study was the least favorable domain judgement. Concerns regarding applicability to the review question (standalone AI assessment of periapical lesions on CBCT) were judged as low, high, or unclear for the patient selection, index test, and reference standard domains. Disagreements were resolved by discussion, and no automation tools were used. Judgements were visualized with robvis [22].

### 2.8. Data Synthesis

Findings were first summarized in a structured narrative synthesis following the Synthesis Without Meta-analysis (SWiM) reporting guideline [23], grouped by AI task: (1) detection, classification, and grading; and (2) lesion segmentation. Within each group, results were described by metric, architecture, unit of analysis, and validation approach. Detection/classification metrics and segmentation metrics were never combined in the same analysis because they address different questions: the former quantify the correct identification of diseased and non-diseased units, whereas DSC quantifies the spatial overlap between predicted and reference lesion masks [24].

### 2.9. Meta-Analysis

Sensitivity and specificity were synthesized jointly with the bivariate random-effects model of Reitsma et al. [25], which accounts for their correlation across studies and for within-study binomial variability, is equivalent to the hierarchical summary receiver operating characteristic (HSROC) model [26] when no covariates are included, and is the approach recommended for diagnostic test accuracy reviews [27]. The model was fitted by restricted maximum likelihood (REML) to the studies that reported, or allowed exact reconstruction of, a complete 2 × 2 table at a single unit of analysis, using the rma.mv function of the metafor package (version 5.0.1) in R 4.5.3 [28]. Summary sensitivity and specificity with 95% confidence intervals (CIs), the between-study variances and their correlation, the diagnostic odds ratio, and a summary receiver operating characteristic (SROC) curve with 95% confidence and prediction regions were derived. Because bivariate models can be unstable when few studies are available [29], the model was refitted (i) adding studies whose 2 × 2 tables could be reconstructed only approximately from reported proportions and (ii) omitting one study at a time.

Because fewer than half of the included studies provided complete 2 × 2 data, each metric was also pooled in a univariate random-effects model of logit-transformed proportions, which allowed every eligible study to contribute and provided descriptive summaries for accuracy, F1-score, and DSC, for which no bivariate counterpart exists. Estimates were entered as event rates with their denominators, logit-transformed, and weighted by inverse variance; within-study variance was derived from the binomial distribution, and between-study variance (τ^2^) was estimated with the DerSimonian–Laird method, as implemented in Comprehensive Meta-Analysis (version 3.3.070; Biostat, Englewood, NJ, USA) [30] and replicated in Python 3.11; the analysis code and dataset are available as Supplementary Materials. For estimates of 0% or 100%, a continuity correction of 0.5 was added to the numbers of events and non-events. Pooled estimates and 95% CIs were back-transformed to the proportion scale, and 95% prediction intervals were calculated on the logit scale using the t distribution with k − 2 degrees of freedom. Because accuracy and F1-score depend on the proportion of lesions in the test set [31] and because the binomial approximation does not reflect the true sampling variance of F1-score or DSC, these pooled values are presented as descriptive summaries and are interpreted with caution. AUC values were not pooled: only three studies reported an AUC, one of them a macro-averaged AUC from a four-class grading task, and the weights assigned by the binomial approximation to an AUC are arbitrary; AUCs are therefore summarized narratively.

### 2.10. Unit of Analysis and Multiplicity

Several studies contributed more than one estimate to a metric, namely tooth- and root-level results, separate ML and DL models, multiple active-learning configurations evaluated on the same test set, and internal and external test sets (Table S3). Treating such estimates as independent would overweight these studies and yield CIs that are too narrow. The primary univariate analysis therefore included one prespecified estimate per study (Section 2.5). Two secondary analyses used all estimates: a single-level model that treats estimates as independent, and a three-level random-effects model with estimates nested within studies, fitted by REML with metafor, which partitions heterogeneity into between-study and within-study components. Two studies that used the same public dataset [32,33] were treated as separate studies in the primary analysis, and the later study was omitted in a sensitivity analysis. The numbers of studies and of estimates are reported for each analysis.

### 2.11. Heterogeneity, Sensitivity Analyses, and Small-Study Effects

Statistical heterogeneity was assessed with Cochran’s Q test and quantified with the I^2^ statistic [34], interpreted according to the Cochrane Handbook: 0–40%, might not be important; 30–60%, moderate; 50–90%, substantial; and 75–100%, considerable [35]. τ^2^ and 95% prediction intervals are reported for each analysis. Robustness was examined with leave-one-study-out analyses, in which all estimates from one study were omitted at a time, and with analyses restricted to studies with verified test-set denominators, excluding the study that reported estimates of 100% [36] and excluding the study that re-used a public dataset [32]. Subgroup analyses and meta-regression were not performed because every analysis included fewer than ten studies in at least one stratum, which is insufficient for an informative investigation of effect modifiers [35]; instead, pooled estimates were compared descriptively between independent evaluations of existing models or commercial platforms and model-development studies, and potential sources of heterogeneity (AI task, architecture, commercial versus in-house system, input data, unit of analysis, validation strategy, reference standard, and test-set size) were tabulated alongside every estimate (Table S3) and alongside the reference-standard characteristics of each study (Table S4). Funnel plots are provided in the Supplementary Materials for transparency only: because every analysis included fewer than ten studies and because funnel-plot asymmetry is a poor indicator of publication bias in diagnostic accuracy reviews [37,38], small-study effects and publication bias were not formally assessed.

### 2.12. Certainty of Evidence

The certainty of the evidence for each pooled outcome was assessed with the GRADE approach for test accuracy [39,40]. Because the included studies were cross-sectional accuracy studies, the evidence started at high certainty and was downgraded by one or two levels for risk of bias, indirectness, inconsistency, imprecision, and publication bias. Risk of bias was judged from the QUADAS-2 assessments of the contributing studies; indirectness from applicability concerns, lesion-enriched or development datasets, and differences in the unit of analysis; inconsistency from I^2^, τ^2^, and the width of the 95% prediction interval or prediction region; and imprecision from the width of the 95% CI. Judgements were made by two reviewers by consensus.

## 3. Results

### 3.1. Study Selection

The database searches identified 241 records (PubMed, *n* = 37; Cochrane Library, *n* = 10; Web of Science, *n* = 71; Scopus, *n* = 74; Google Scholar, *n* = 49); the trial registries yielded no records. After the removal of 53 duplicates, 188 titles and abstracts were screened, and 157 records were excluded. Of the 31 full-text articles assessed, 13 were excluded because they did not use CBCT (*n* = 6), did not evaluate AI (*n* = 4), or were review articles (*n* = 3) (Table S2). Four further reports were identified from websites (*n* = 3) and organizations (*n* = 1); all were retrieved and assessed, and none met the inclusion criteria (population or setting not eligible, *n* = 3; index test not eligible, *n* = 1). Citation searching identified no additional eligible studies. Eighteen studies were included [32,33,36,41,42,43,44,45,46,47,48,49,50,51,52,53,54,55] (Figure 1).

### 3.2. Characteristics of Included Studies

The 18 studies were published between 2009 and 2025 and were conducted in ten countries: the United States (five studies), China and Turkey (three each), and Australia, Austria, Brazil, Egypt, India, Russia, and the United Kingdom (one each) (Table 2 and Table 3). Eleven studies addressed lesion detection, classification, or grading [32,33,36,41,42,44,45,48,49,53,55]; four primarily addressed segmentation, three of which also reported lesion-level detection [46,47,51,52]; two addressed both detection and segmentation [50,54]; and one used AI to localize the posterior superior alveolar artery in patients with periapical lesions [43]. Five studies evaluated commercial platforms (Diagnocat in four [41,45,48,53] and eVol DXS in one [43]), and 13 evaluated in-house models, including CNN-based (U-Net, ResNet, VGG16, DenseNet-121, and Mask R-CNN), transformer-based (Swin-UNETR), and hybrid (PAINet) architectures.

Only one study externally validated a newly developed model on independent multicenter datasets [50]; four independently evaluated an existing model or commercial platform on new clinical data [41,42,45,53], and the remainder relied on internal hold-out test sets or cross-validation. The reference standard was established by two or more readers with a described consensus procedure in ten studies [41,42,43,46,47,48,50,51,54,55], by two readers without a described consensus procedure in one [36], by a single reader in two [33,53], by semi-automated software segmentation (Mimics Research v21.0.0.406; Materialise NV, Leuven, Belgium) in one [45], by the pre-existing labels of a public dataset in one [32], and by expert diagnosis supplemented by histopathology in one [49]; the number and expertise of readers were not reported in two studies [44,52]. Blinding of the reference standard to the AI output was explicitly reported, or ensured by establishing the reference standard before AI was used, in three studies [41,42,48] (Table 3; full details in Table S4). Akalin and Özkan used the public dataset released by Calazans et al. [32,33].

### 3.3. Risk of Bias and Applicability

**Figure 2:**
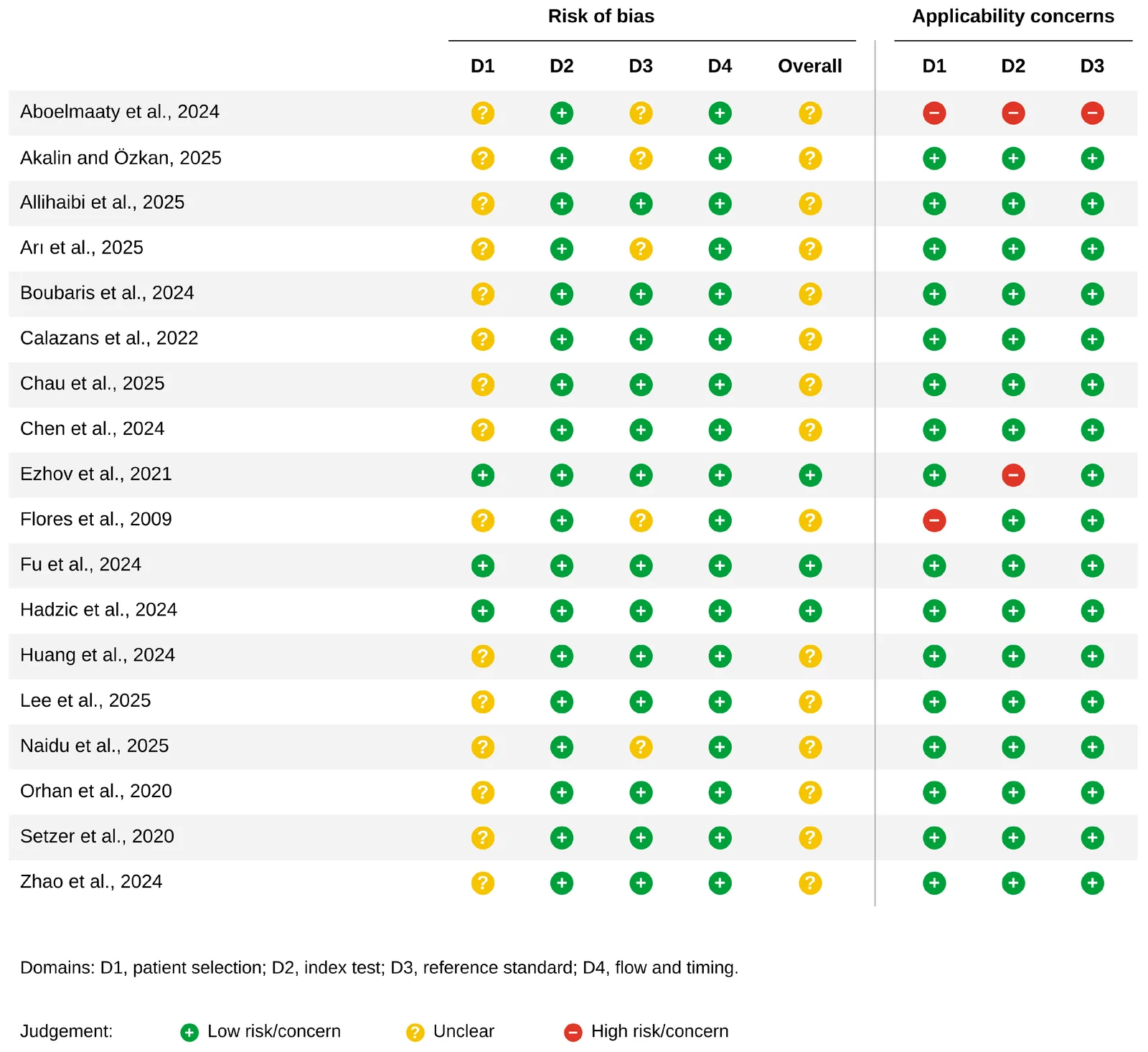
QUADAS-2 risk-of-bias and applicability judgements for each included study (traffic-light plot). D1, patient selection; D2, index test; D3, reference standard; D4, flow and timing; QUADAS-2, Quality Assessment of Diagnostic Accuracy Studies-2. Data from [32,33,36,41,42,43,44,45,46,47,48,49,50,51,52,53,54,55].

**Figure 3:**
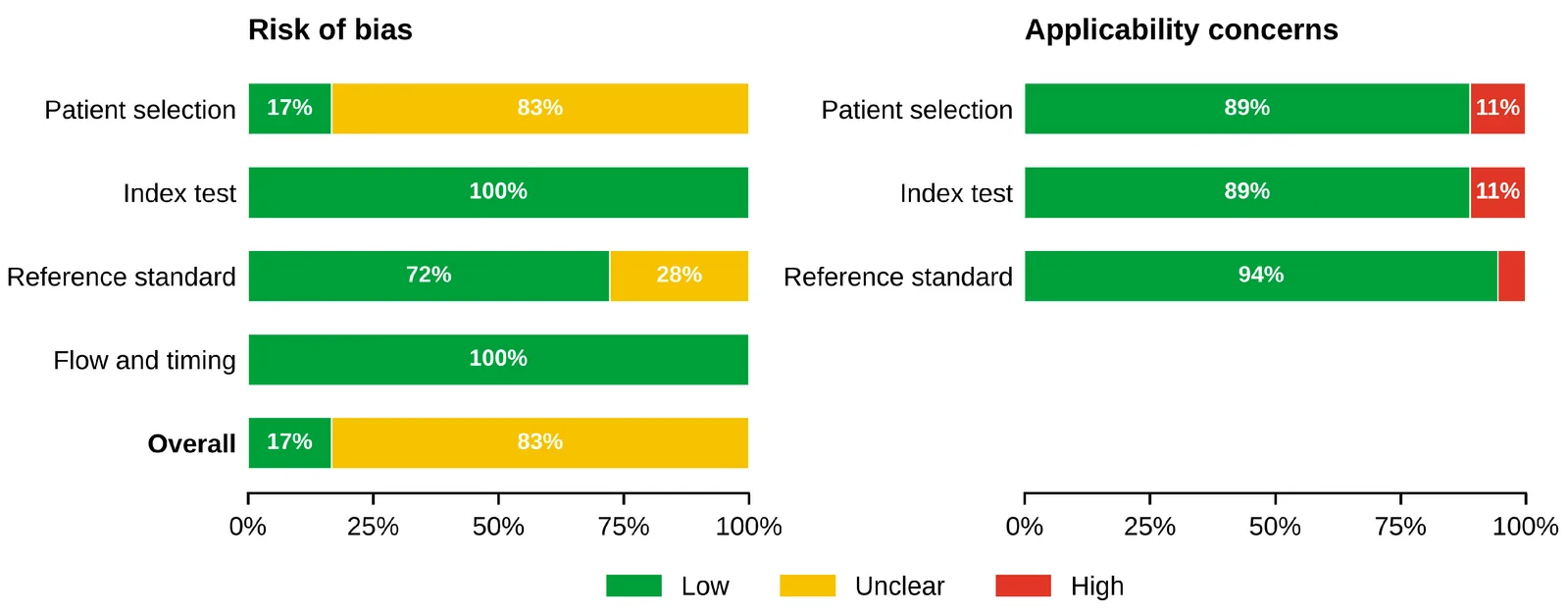
Summary of QUADAS-2 risk-of-bias and applicability judgements across the included studies (percentage of studies). QUADAS-2, Quality Assessment of Diagnostic Accuracy Studies-2.

Three studies were at low risk of bias in all domains [42,48,50], and 15 were rated unclear in at least one domain (Figure 2 and Figure 3; Table 4). Concerns arose in patient selection (15 studies), reflecting non-consecutive or curated sampling and lesion-enriched datasets, and in the reference standard (five studies [32,36,43,44,49]). All studies were rated at low risk in the index-test and flow-and-timing domains, and no domain was rated at high risk; this pattern partly reflects incomplete reporting rather than demonstrated methodological quality. High applicability concerns were identified for three studies: Aboelmaaty et al., in which AI was not applied to lesion assessment [43]; Flores et al., which addressed the differential diagnosis of established lesions [49]; and Ezhov et al., whose primary lesion outcome was AI-assisted reader performance [48].

### 3.4. Narrative Synthesis

The narrative synthesis is organized by outcome theme. Per-study descriptions of every reported metric, together with the model configurations, test sets, and denominators from which each value was taken, are provided in Supplementary Text S1 and Tables S3 and S7; the main text summarizes the pattern across studies.

#### 3.4.1. Sensitivity and Specificity for Lesion Detection

Fourteen studies reported standalone sensitivity for the presence of a periapical lesion at a defined unit of analysis. Sensitivity exceeded 90% in nine of them, including the three independent evaluations of commercial platforms (Diagnocat: 93.8% at tooth level in molars [41]; 92.6% at root level when CBCT periapical volume index (CBCTPAVI) grading was dichotomized [45]; 92.8% in a lesion-positive sample [53]) and the 3D models evaluated on held-out or external data (PAL-Net 97.2% on an independent test set and 87.7–94.0% across three external centers [50]; Swin-UNETR 100% [47]; CBCT-SAM 98.9% [46]). Sensitivity was 83.8–86.7% for a CNN evaluated on a clinically representative dataset in which 300 of 2947 teeth had lesions [42], for the standalone periapical-lesion module of a multi-condition platform [48], and for a Bayesian U-Net trained with active learning on 26 slices [51], and it was below 70% for the two-dimensional slice classifiers trained on the Federal University of Pernambuco (UFPE) dataset (64.5% [33] and 58.0% [32]). Within studies, sensitivity fell with lesion size (very small lesions with a periapical index score of 1 [42]; indices 1–2 not detected [45]) and with the training strategy (32% for the Bayesian U-Net without active learning [51]).

Eleven studies reported standalone specificity. It was lowest for tooth-level detection in root-filled and non-root-filled molars (65.2%; 79.9% at root level) [41] and for the slice classifiers (63.0% [32] and 76.3% [33]), intermediate on the clinically representative dataset (84.2%) [42] and for the U-Net- and active-learning-based models (87.5–90.3%) [51,54], and 92% or higher for Swin-UNETR [47], PAL-Net (96.2% on the independent test set, reconstructed from the reported sensitivity and positive predictive value) [50], Diagnocat in the CBCTPAVI study (96.7%) [45], and the periapical-lesion module of Ezhov et al. (99.5%) [48]. An ML classifier reported 100% sensitivity and specificity on a 96-scan test set in which every scan contained a lesion of one of three types, so that these values describe lesion-type classification rather than detection [36]. Specificity computed across voxels, most of which are background (99.9% [46]), is not comparable with tooth- or lesion-level specificity and was not pooled.

#### 3.4.2. Accuracy, Precision, and F1-Score

Binary detection accuracy was 58.6% [32] and 70.0% [33] for slice classifiers, 79.1% (tooth level) and 82.6% (root level) for Diagnocat in molars [41], 84.5% on the clinically representative dataset [42], 87.5–90.2% for small-sample U-Net-based models [51,54], 93.4% for dichotomized CBCTPAVI grading [45] and 94.0% for Swin-UNETR [47]. Four-class grading accuracy was 93.3% for PAINet [55], and a linear discriminant analysis (LDA)-AdaBoost classifier differentiated cysts from granulomas with 94.1% accuracy [49], a differential-diagnosis task that was not pooled. Precision (PPV) was 0.94 for PAINet [55], 0.95 for Diagnocat in a lesion-positive sample [53], 0.91 for the regularized VGG16 [32], 0.87 for a U-Net-based model [54], 0.79 for OAK-SSL on small lesions [52], 0.76–0.92 for PAL-Net across test sets [50], 0.76 for DenseNet-121 [33], and 0.72–0.76 for Diagnocat in molars [41]. F1-scores were 0.93 for PAINet [55] and Diagnocat [53], 0.87 for periapical lesions within a 15-condition Mask R-CNN model [44], 0.79–0.93 for PAL-Net [50], 0.81 for Diagnocat in molars [41], and 0.70–0.71 for the slice classifiers [32,33]. In the one study that reported postoperative scans separately, PPV and F1-score fell to 54% and 67% in root-filled molars [41].

#### 3.4.3. Discrimination (Area Under the Curve)

Three studies reported an AUC. In the independent evaluation of a commercial platform in molars, the AUC was 0.76 at tooth level and 0.79 at root level [41]. PAL-Net reached 0.98 in internal cross-validation, 0.97 on the independent internal test set, and 0.93–0.96 on three external datasets [50]. PAINet reached a macro-averaged AUC of 0.997 in a four-class grading task, compared with 0.994 and 0.990 for VOLO-D1 and Swin Transformer-Large comparators [55]. These values were not pooled (Section 2.9): the three studies differ in task (binary detection versus four-class grading), unit of analysis, and validation design, and the development-study AUCs were derived from curated datasets, whereas the lowest AUC came from the only independent clinical evaluation.

#### 3.4.4. Agreement with Clinicians and Reader Studies

PAINet showed almost perfect agreement with expert grading (κ = 0.911) [55]. Diagnocat volumetric measurements agreed closely with manual segmentation (Bland–Altman mean difference, 0.15 mm; 95% limits of agreement, −1.21 to 1.52) [53], and AI and semi-automated CBCTPAVI scores agreed exactly for 84.4% of roots, with no lesion classified as index 1 or 2 by AI [45]. PAL-Net performed comparably to or better than the participating dentists on the study datasets (AUC 0.98 versus 0.93 for the most experienced dentist) and reduced reading time by 32–69 min per 100 scans [50]. In a reader study of 24 dentists, aggregate sensitivity across all conditions increased from 76.7% without to 85.4% with AI assistance [48]; this reflects AI-assisted reader performance rather than standalone AI performance. No study compared AI and clinicians under a common protocol that would allow a pooled comparison.

#### 3.4.5. Lesion Segmentation

Six studies reported lesion DSC, which quantifies spatial overlap rather than detection. Values were 0.85 for a pretrained Swin-UNETR [47], 0.87 (internal cross-validation), 0.81 (independent test set), and 0.80–0.90 (external datasets) for PAL-Net [50], 0.70 ± 0.19 for CBCT-SAM [46], 0.67 for true lesions (0.52 including false negatives) for a U-Net-based model [54], 0.65 for a semi-supervised model evaluated on volumes with small lesions [52], and 0.50 for Bayesian U-Nets trained with active learning on 26 slices (0.29 without active learning) [51]. Lesion DSC was consistently lower than DSC for teeth and bone within the same studies [47,51,52,54], reflecting the irregular boundaries and small volume of periapical lesions.

#### 3.4.6. Other Applications

One study used a commercial platform to localize the posterior superior alveolar artery (identified in all scans) and found that sinus mucosal thickening was more frequent in lesion-bearing sites when the artery lay beneath the sinus membrane (62.5% vs. 8.6%) [43]; lesion detection itself was not evaluated, and the study was retained for completeness of the narrative synthesis only.

### 3.5. Meta-Analysis

Five metrics were pooled, and AUC was summarized narratively (Section 3.4.3). Table 5 presents the primary estimates, i.e., the bivariate model for sensitivity and specificity and the univariate models with one estimate per study for all metrics; the single-level and three-level analyses of all estimates, the other sensitivity analyses, and the leave-one-study-out analyses are given in Tables S5 and S6, and funnel plots in Figure S1. Per-estimate data, with their denominators and provenance, are listed in Table S7.

#### 3.5.1. Bivariate Synthesis of Sensitivity and Specificity

**Figure 4:**
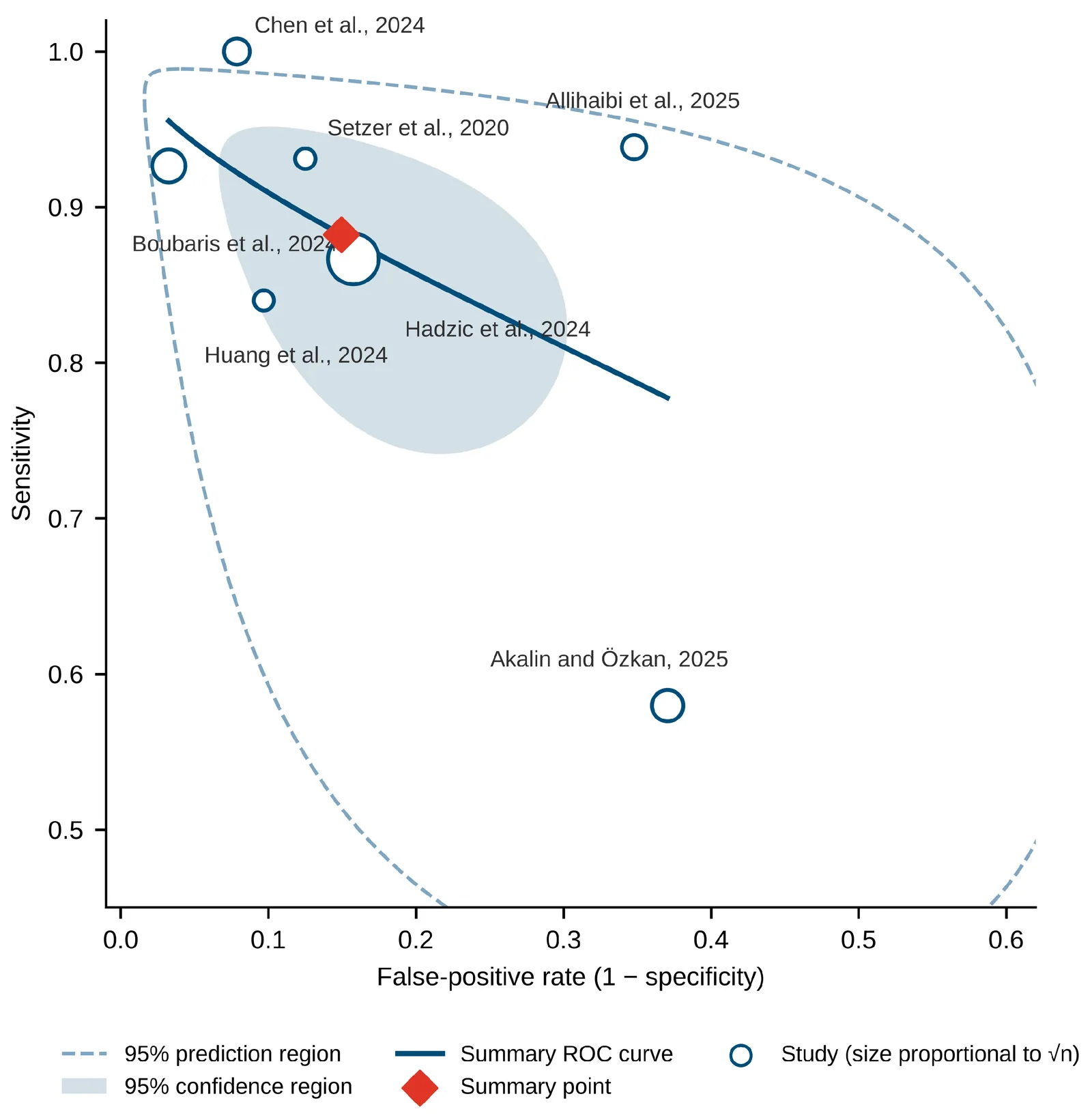
Summary receiver operating characteristic (SROC) plot of the bivariate random-effects model for the seven studies with complete 2 × 2 data. Circles, study estimates (area proportional to the square root of the number of units); diamond, summary point (sensitivity 88.2%, specificity 85.1%); shaded area, 95% confidence region; dashed line, 95% prediction region; solid line, SROC curve. Data from [32,41,42,45,47,51,54].

Seven studies reported or allowed exact reconstruction of a complete 2 × 2 table at a single unit of analysis [32,41,42,45,47,51,54], together comprising 1214 lesion-positive and 3065 lesion-negative units. The bivariate model converged and gave a summary sensitivity of 88.2% (95% CI: 77.6–94.2%) and a summary specificity of 85.1% (73.4–92.1%), with a diagnostic odds ratio of 43 (Figure 4). Between-study variances were large on the logit scale (τ^2^ = 0.84 for sensitivity and 0.80 for specificity), and their correlation was positive (ρ = 0.62), indicating that studies with higher sensitivity also tended to have higher specificity rather than a threshold trade-off; the 95% prediction region was correspondingly wide (sensitivity 51–98%, specificity 46–97%). Adding the three studies whose 2 × 2 tables could be reconstructed only approximately [33,48,50] gave a summary sensitivity of 86.7% (77.3–92.6%) and specificity of 89.2% (78.9–94.8%). In leave-one-study-out analyses, summary sensitivity ranged from 86.4% (omitting Chen et al. [47]) to 90.5% (omitting Akalin and Özkan [32]) and specificity from 81.8% (omitting Boubaris et al. [45]) to 87.8% (omitting Akalin and Özkan [32]) (Table S6). When the original bivariate model was restricted to the two studies that reported complete 2 × 2 data directly [41,42], the between-study correlation was estimated at −1, confirming that it could not be fitted reliably with two studies.

#### 3.5.2. Univariate Synthesis of Detection and Classification Metrics

**Figure 5:**
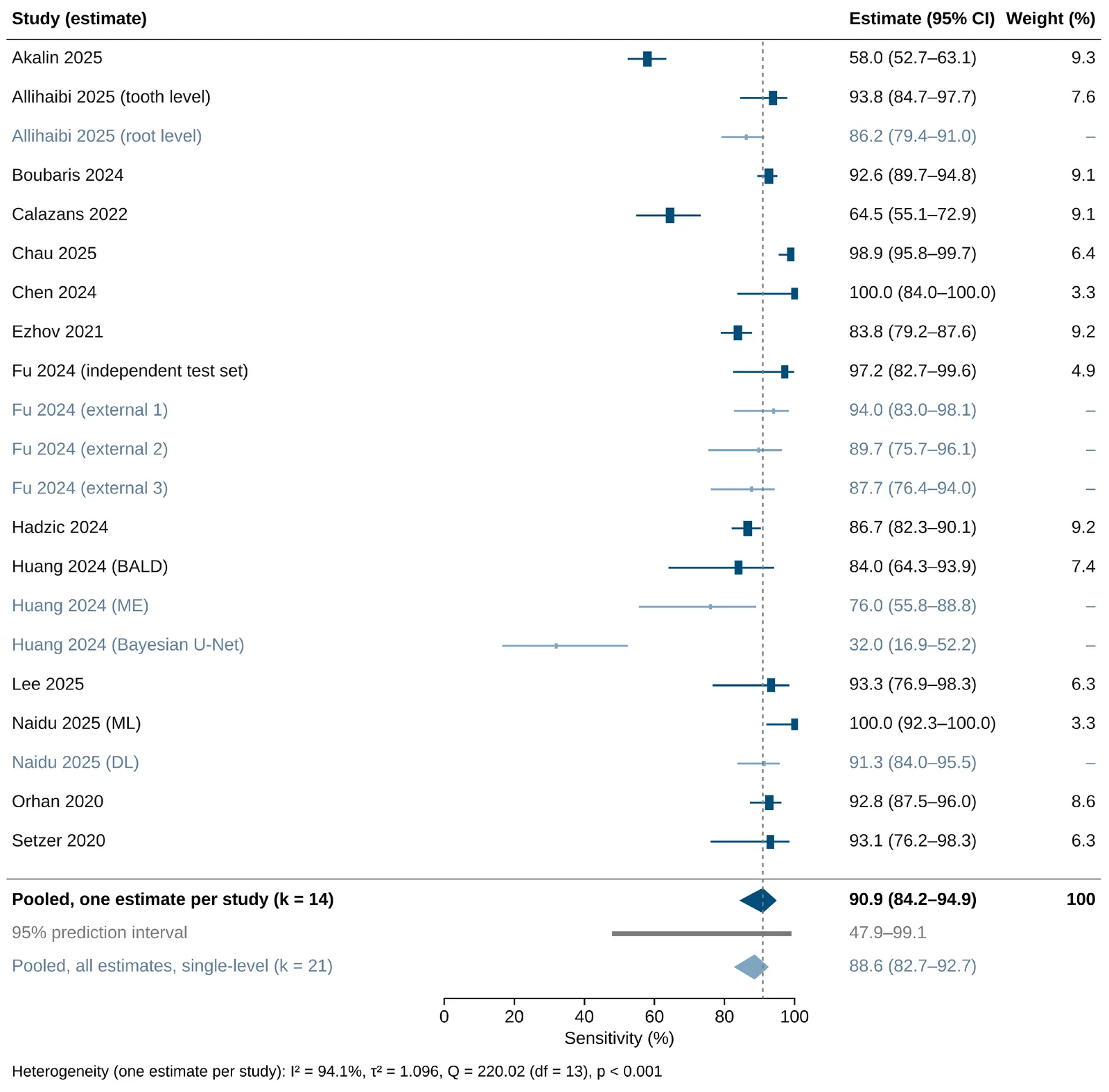
Forest plot of the sensitivity of AI models for periapical lesion detection on CBCT. Dark squares, primary estimate of each study (one per study), sized by random-effects weight; light squares, secondary estimates from the same studies (other unit of analysis, model configuration, or external dataset), which contribute only to the all-estimates analyses; horizontal lines, 95% confidence intervals (CIs); dark diamond, pooled estimate with one estimate per study; grey bar, its 95% prediction interval; light diamond, single-level pooled estimate of all estimates. AI, artificial intelligence; BALD, Bayesian active learning by disagreement; CBCT, cone-beam computed tomography; ME, maximum entropy; ML, machine learning; DL, deep learning. Data from [32,33,36,41,42,45,46,47,48,50,51,52,53,54].

Sensitivity. Fourteen studies contributed one estimate each (21 estimates in total; Figure 5) [32,33,36,41,42,45,46,47,48,50,51,52,53,54]. The pooled sensitivity was 90.9% (95% CI: 84.2–94.9%), with considerable heterogeneity (I^2^ = 94.1%; Q = 220.0, df = 13, *p* < 0.001; τ^2^ = 1.10) and a 95% prediction interval of 47.9–99.1%. Individual estimates ranged from 58.0% for a regularized VGG16 slice classifier [32] to 100% for Swin-UNETR [47] and for an ML classifier [36]. Pooling all 21 estimates gave 88.6% (82.7–92.7%) in the single-level model and 89.0% (81.7–93.6%) in the three-level model, in which between-study and within-study variance components were of similar size (0.60 and 0.61). In leave-one-study-out analyses, the pooled sensitivity ranged from 89.4% (omitting Chau et al. [46]) to 92.3% (omitting Calazans et al. [33]) (Table S6).

**Figure 6:**
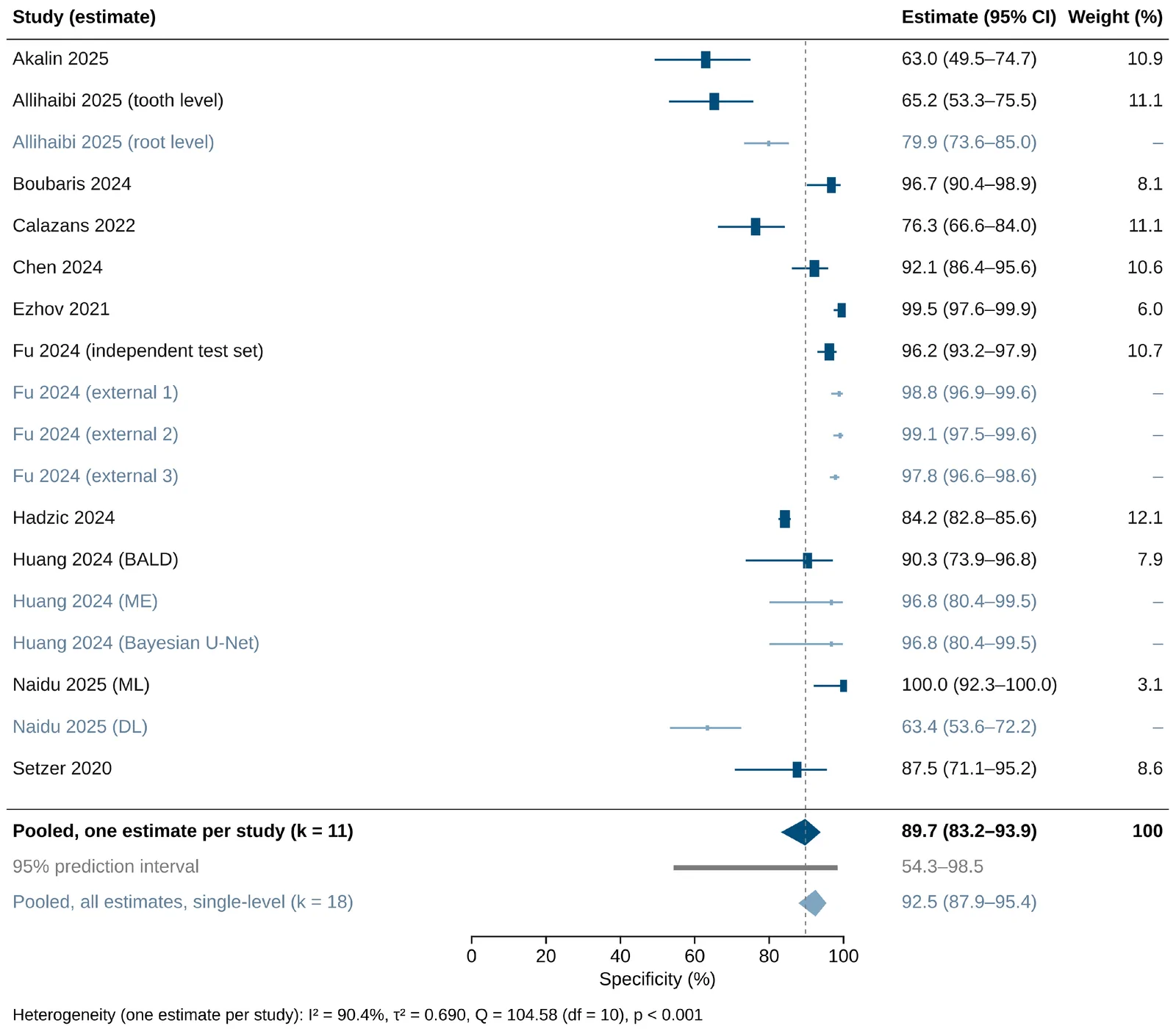
Forest plot of the specificity of AI models for periapical lesion detection on CBCT. Symbols as in Figure 5. AI, artificial intelligence; BALD, Bayesian active learning by disagreement; CBCT, cone-beam computed tomography; DL, deep learning; ME, maximum entropy; ML, machine learning. Data from [32,33,36,41,42,45,47,48,50,51,54].

Specificity. Eleven studies contributed (18 estimates; Figure 6) [32,33,36,41,42,45,47,48,50,51,54]. The pooled specificity was 89.7% (95% CI: 83.2–93.9%), with considerable heterogeneity (I^2^ = 90.4%; Q = 104.6, df = 10, *p* < 0.001; τ^2^ = 0.69) and a 95% prediction interval of 54.3–98.5%. The lowest estimates came from the slice classifier [32] (63.0%) and from tooth-level detection in molars [41] (65.2%). The single-level and three-level analyses of all estimates gave 92.5% (87.9–95.4%) and 91.0% (81.8–95.8%), respectively; in the three-level model, between-study variance (1.21) exceeded within-study variance (0.67), reflecting the larger differences between studies than between configurations of the same model. Leave-one-study-out estimates ranged from 87.4% (omitting Ezhov et al. [48]) to 91.4% (omitting Akalin and Özkan [32], Allihaibi et al. [41], or Hadzic et al. [42]).

**Figure 7:**
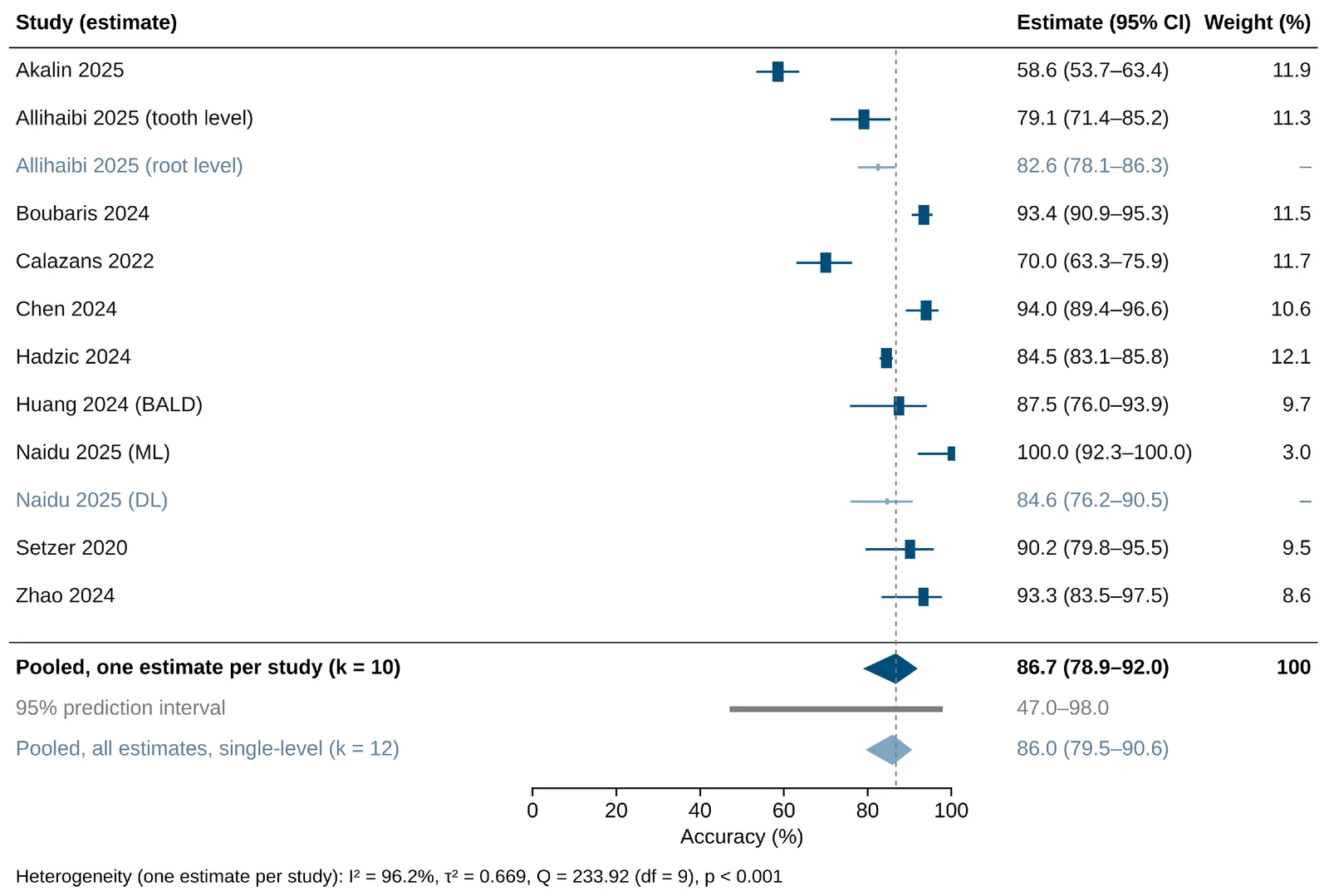
Forest plot of the accuracy of AI models for periapical lesion detection and classification on CBCT. Symbols as in Figure 5. AI, artificial intelligence; BALD, Bayesian active learning by disagreement; CBCT, cone-beam computed tomography; DL, deep learning; ME, maximum entropy; ML, machine learning. Data from [32,33,36,41,42,45,47,51,54,55].

Accuracy. Ten studies contributed (12 estimates; Figure 7) [32,33,36,41,42,45,47,51,54,55]. The pooled accuracy was 86.7% (95% CI: 78.9–92.0%), with considerable heterogeneity (I^2^ = 96.2%; Q = 233.9, df = 9, *p* < 0.001; τ^2^ = 0.67) and a 95% prediction interval of 47.0–98.0%. All-estimates analyses gave 86.0% (79.5–90.6%) and 85.7% (78.3–90.9%), and leave-one-study-out estimates ranged from 85.4% to 88.4%.

**Figure 8:**
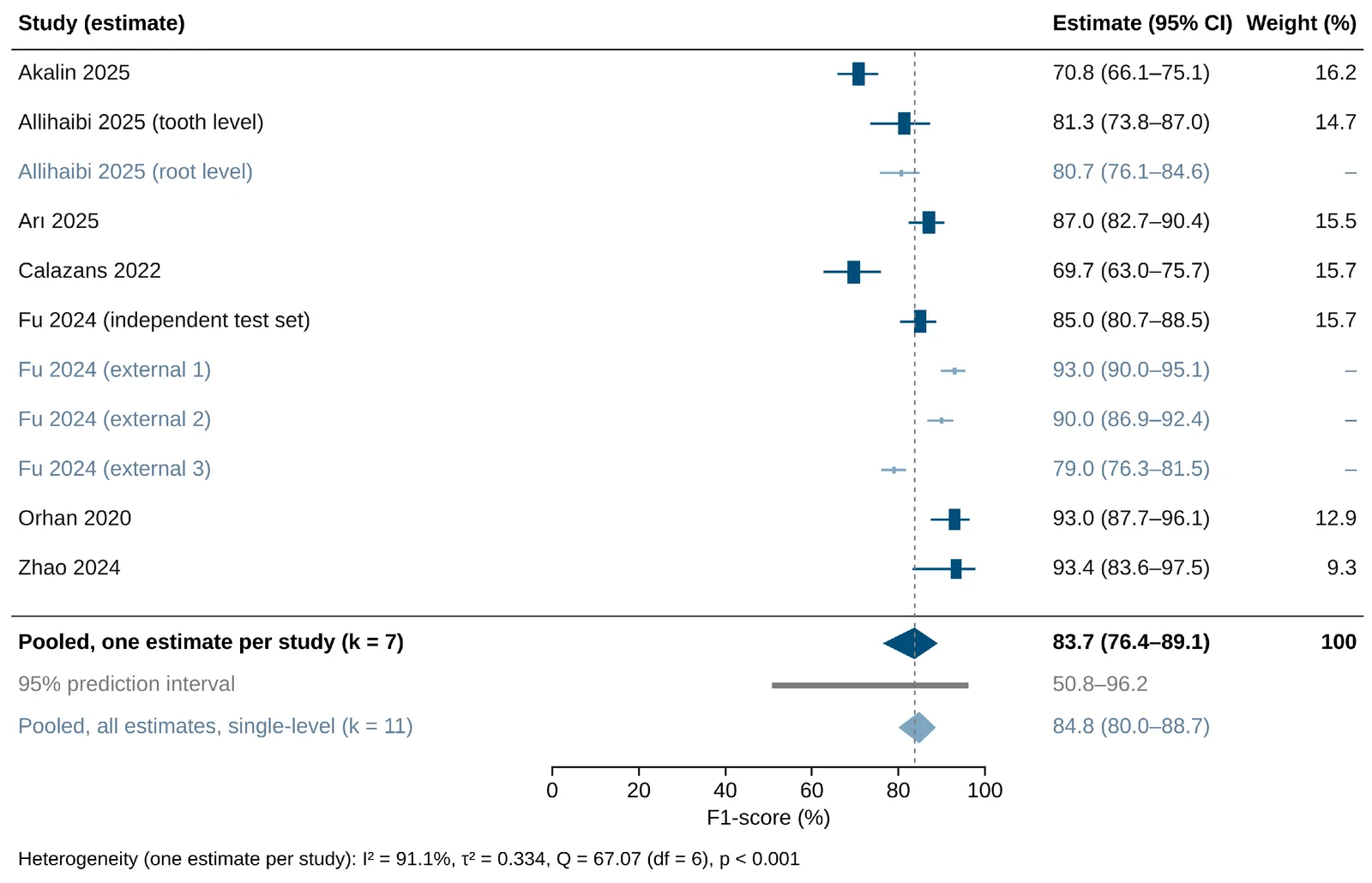
Forest plot of the F1-score of AI models for periapical lesion detection and classification on CBCT. Symbols as in Figure 5. AI, artificial intelligence; CBCT, cone-beam computed tomography. Data from [32,33,41,44,50,53,55].

F1-score. Seven studies contributed (11 estimates; Figure 8) [32,33,41,44,50,53,55]. The pooled F1-score was 83.7% (95% CI: 76.4–89.1%), with considerable heterogeneity (I^2^ = 91.1%; Q = 67.1, df = 6, *p* < 0.001; τ^2^ = 0.33) and a 95% prediction interval of 50.8–96.2%. All-estimates analyses gave 84.8% (80.0–88.7%) and 84.5% (77.7–89.5%), and leave-one-study-out estimates ranged from 81.5% (omitting Orhan et al. [53]) to 85.7% (omitting Calazans et al. [33]).

#### 3.5.3. Segmentation

**Figure 9:**
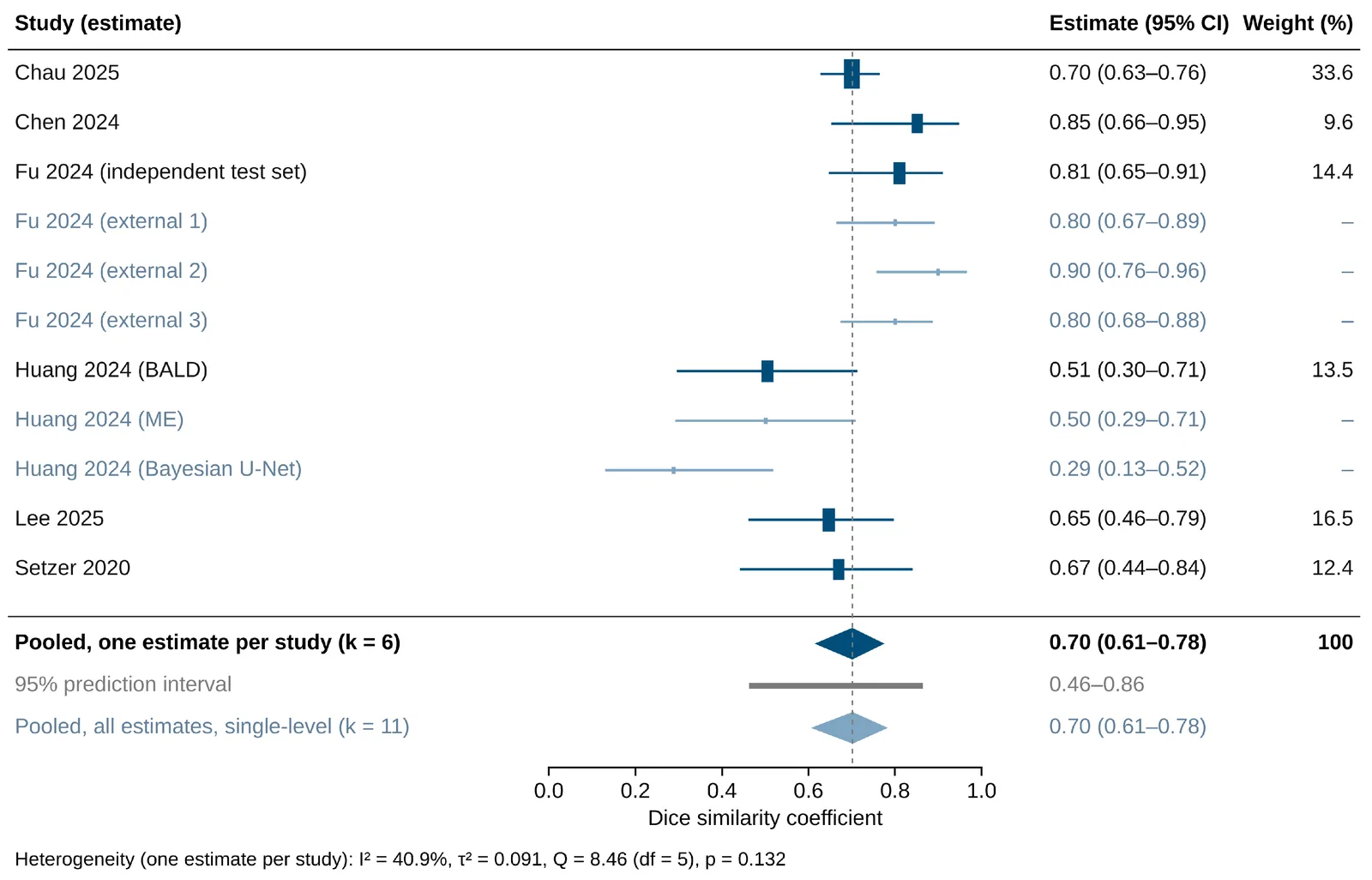
Forest plot of the Dice similarity coefficient (DSC) of AI models for periapical lesion segmentation on CBCT. Symbols as in Figure 5. AI, artificial intelligence; BALD, Bayesian active learning by disagreement; CBCT, cone-beam computed tomography; ME, maximum entropy. Data from [46,47,50,51,52,54].

Six studies contributed one lesion DSC estimate each (11 estimates in total; Figure 9) [46,47,50,51,52,54]. The pooled DSC was 0.70 (95% CI: 0.61–0.78), with moderate heterogeneity (I^2^ = 40.9%; Q = 8.46, df = 5, *p* = 0.132; τ^2^ = 0.09) and a 95% prediction interval of 0.46–0.86. All-estimates analyses gave 0.70 (0.61–0.78) and 0.70 (0.56–0.80); heterogeneity among all 11 estimates was substantial (I^2^ = 72.2%) and, in the three-level model, was attributable entirely to differences between studies. Leave-one-study-out estimates ranged from 0.68 (omitting Chen et al. [47] or Fu et al. [50]) to 0.72 (omitting Huang et al. [51]).

#### 3.5.4. Sensitivity Analyses and Exploration of Heterogeneity

The pooled estimates were robust to the analytical choices examined (Table S5). Excluding the study that reported estimates of 100% [36] changed the pooled sensitivity, specificity, and accuracy by approximately one percentage point (90.0%, 88.7%, and 85.5%, respectively). Restricting the analyses to studies with verified test-set denominators gave a pooled sensitivity of 92.3% (10 studies), specificity of 86.5% (eight studies), accuracy of 84.3% (eight studies), and F1-score of 83.0% (six studies). Omitting the study that re-used the UFPE dataset [32] increased the pooled sensitivity to 91.7% and reduced its heterogeneity (I^2^ = 86.7%), while increasing specificity, accuracy, and F1-score to 91.4%, 88.4%, and 85.6%, respectively; the same study was the most influential in the bivariate model (Section 3.5.1).

Descriptively, several sources of heterogeneity were apparent (Table S3). First, the four independent evaluations of existing models or commercial platforms [41,42,45,53] had a pooled sensitivity of 91.3% (95% CI: 87.1–94.2%; I^2^ = 66.7%) that was similar to that of development studies (90.3%; 81.3–95.2%), whereas their pooled specificity (85.1%; 67.9–93.9%; three studies) was lower than that of development studies (92.4%; 82.5–96.9%; eight studies), and the lowest AUC and one of the two lowest specificities were observed in the only independent evaluation restricted to molars [41]. Second, the two studies with the lowest sensitivity and accuracy both classified two-dimensional slices of the same public dataset of small lesions [32,33], whereas models operating on three-dimensional volumes [46,47,50] reported the highest values. Third, the unit of analysis ranged from image sections [32,33,55] and scans [36] to teeth [41,42,48,50], roots [45,47,51,54], and lesions [46,53]; within one study, specificity was 65.2% at tooth level and 79.9% at root level [41]. Fourth, estimates varied within studies according to model type [36], training strategy [51], and test set [50]. Fifth, test sets ranged from 20 slices [51] and 25 volumes [47] to 2947 teeth [42], and reference standards ranged from a single observer or pre-existing dataset labels to multi-reader consensus (Table S4). Because each analysis included fewer than ten studies in at least one stratum, the independent contribution of these factors could not be quantified.

### 3.6. Certainty of Evidence

The certainty of the evidence was very low for every pooled outcome (Table 6). All analyses were downgraded for risk of bias, because most contributing studies were rated unclear for patient selection and for indirectness, because the evidence came mainly from lesion-enriched development datasets with heterogeneous units of analysis. The detection metrics were downgraded by two levels for inconsistency, given the considerable heterogeneity and prediction intervals or regions extending below 55%, and the bivariate specificity was additionally downgraded for imprecision; lesion DSC was downgraded by one level for inconsistency because its prediction interval was wide despite moderate statistical heterogeneity.

## 4. Discussion

### 4.1. Principal Findings

This review synthesized 18 studies of AI for periapical lesion assessment on CBCT. In the bivariate synthesis of the seven studies with complete 2 × 2 data, summary sensitivity and specificity were 88.2% and 85.1%; in univariate analyses that included one estimate from every eligible study, pooled sensitivity, specificity, and accuracy were 90.9%, 89.7%, and 86.7%, and the pooled F1-score was 83.7%. For segmentation, the pooled lesion DSC was 0.70. These estimates indicate promising performance under the conditions of the included studies. However, they are accompanied by considerable heterogeneity, prediction intervals that extend below 55% for every detection metric, frequent concerns about patient selection, incompletely reported reference standards, and a predominance of retrospective, internally validated development studies. They should therefore be regarded as an optimistic summary of reported research performance rather than as the performance expected in routine practice.

### 4.2. Interpretation of Detection and Classification Metrics

Sensitivity and specificity are the most informative metrics for clinical translation because, unlike accuracy, precision, and F1-score, they do not depend directly on the prevalence of lesions in the test set [31]. High sensitivity is desirable when AI is used to flag possible lesions for clinician review. Nevertheless, diagnostic accuracy alone does not show that AI use reduces disease progression, prevents complications, or improves patient outcomes; these effects require evaluation in studies of clinical impact [56,57]. The summary specificity suggests an acceptable false-positive rate under study conditions, but its wide CI and prediction interval, and the lower specificity observed in the independent evaluation of root-filled and non-root-filled molars (65.2% at tooth level) [41] and in the two-dimensional slice classifiers [32,33], indicate that false-positive findings may be considerably more frequent in clinically complex teeth and in small lesions, where they could prompt unnecessary retreatment or referral. The positive between-study correlation of sensitivity and specificity in the bivariate model suggests that differences between studies reflect overall model and dataset quality more than different operating thresholds. Because many datasets were lesion-enriched, accuracy and F1-score may overstate performance in populations with a lower prevalence of disease [31]. The three reported AUCs were not pooled: the independent clinical evaluation yielded 0.76–0.79 [41], whereas values of 0.93–0.997 came from development datasets [50,55], and a pooled value would have been dominated by whichever study was assigned the largest denominator rather than by the quality of the evidence.

Sadr et al. reported high pooled performance for DL detection of periapical radiolucencies across imaging modalities, together with notable risk-of-bias concerns [16], and a recent narrative review likewise described generally high performance across modalities [17]. The present review extends this evidence by focusing on CBCT, separating detection from segmentation, synthesizing sensitivity and specificity jointly where the data allow, and applying QUADAS-2 with AI-specific signaling questions. It identifies a similar dependence on curated, internally validated datasets, and its prediction intervals indicate that performance in a new setting may differ considerably from the pooled estimates.

### 4.3. Interpretation of Segmentation Performance

The pooled DSC of 0.70 indicates moderate spatial overlap between AI-predicted and reference lesion boundaries; it does not represent the proportion of lesions correctly detected or classified [24]. Lesion DSC was consistently lower than DSC for teeth or bone within the same studies [47,51,52,54], reflecting irregular borders, small lesion volumes, partial-volume effects, and inter-annotator variability in delineation; DSC is also particularly penalizing for small structures [24]. Higher values were reported for transformer-based and 3D models trained on larger datasets [47,50], but these came from development settings, and the external DSC of the only externally validated model (0.80–0.90) was close to its internal value [50]. Accurate segmentation is relevant to the volumetric monitoring of healing [45,53], but the level of overlap that is clinically meaningful has not been established.

### 4.4. Model Architectures and Comparison with Clinicians

Transformer-based, hybrid, and 3D models reported the highest values [46,47,50,55]; however, because most were evaluated in development studies with internal validation, their apparent advantage may partly reflect optimistic estimation rather than generalizable superiority [56]. Only two studies compared AI with clinicians, using different designs [48,50], and no pooled comparison was possible. The present results therefore do not establish that AI is equivalent or non-inferior to experienced clinicians; claims of expert-level performance are common in the DL literature but are rarely supported by adequately designed comparative studies [57].

The included studies used task-specific models trained on annotated CBCT data. Since the search date, general-purpose foundation models have begun to be applied to dental imaging without task-specific training: in a zero-shot evaluation of three vision-language models on sagittal CBCT slices of the mandibular condyle, the best model identified condylar osseous changes with 90.3% accuracy (sensitivity 84.4%, specificity 95.0%) against dual-radiologist consensus, with false negatives in subtle early changes as the dominant error [58], and large language models are being used to generate educational material in dentistry, where human review of the generated content remains necessary to guarantee accuracy [59]. These approaches have not yet been evaluated for periapical lesions on CBCT. Their accessibility through web interfaces could lower the barrier to decision support in general practice, but the conservative diagnostic tendency, closed-source architectures, and lack of fine-tuning reported for current vision-language models [58] mean that they would need the same prospective: externally validated evaluation against a rigorous reference standard that this review identifies as lacking for task-specific models.

### 4.5. Strengths and Limitations

The strengths of this review include a registered protocol, a multi-database search with full reporting of strategies, the use of QUADAS-2 with AI-specific signaling questions, the separation of detection/classification from segmentation outcomes, verification of every pooled value and denominator against the full text of each study, joint synthesis of sensitivity and specificity with a bivariate model, one-estimate-per-study and multilevel analyses that respect the clustering of estimates within studies, transparent reporting of the number of studies and estimates, τ^2^, and prediction intervals for each analysis, and a GRADE assessment of the certainty of the evidence.

The evidence base has important limitations. Most studies used small, retrospective, and often lesion-enriched datasets, which may introduce spectrum bias and inflate performance. Most relied on internal validation; only one reported external multicenter validation of a newly developed model [50]. Reference standards were heterogeneous and incompletely described, particularly regarding the number and expertise of annotators, consensus procedures, and blinding (Table S4); a single observer or pre-existing dataset labels served as the reference standard in three studies [32,33,53] and a semi-automated software measurement in one [45], and blinding to AI output was documented in only three [41,42,48]. Two studies used the same public dataset [32,33], so their results are not fully independent. CBCT acquisition parameters, lesion size, and root-filling status were inconsistently reported, and the unit of analysis varied from image sections to teeth, roots, lesions, and voxels.

The review itself also has limitations. First, the bivariate model could be fitted to only seven studies with complete 2 × 2 data, and the univariate analyses, which include every study, ignore the correlation between sensitivity and specificity and threshold effects; the two approaches gave consistent results. Second, the test-set denominators of four estimates could be reconstructed only approximately (Table S7), one study was available only as an abstract [44], and the denominators of one study were not reported [36]; analyses restricted to verified denominators gave similar estimates. Third, the binomial approximation used for F1-score and DSC does not reflect their true sampling variance, and the weights in these analyses depend on the test-set sizes used to approximate it. Fourth, two estimates were derived rather than reported directly (binary detection from a grading confusion matrix [45] and specificity from sensitivity and positive predictive value [50]). Fifth, the authors of the primary studies were not contacted, so unreported data could not be retrieved. Sixth, the small number of studies per analysis precluded subgroup analysis and meta-regression, and publication bias could not be assessed; because studies with unfavorable results may remain unpublished, the pooled estimates are more likely to overestimate than to underestimate performance. Seventh, the absence of high risk-of-bias judgements partly reflects incomplete reporting in the primary studies rather than demonstrated methodological quality.

### 4.6. Implications for Practice and Research

At present, AI-based CBCT analysis may be considered a decision-support tool that flags possible lesions for verification by the clinician; it should complement, not replace, clinical and radiological judgement [60]. The included studies also identify the situations in which current models fail. Sensitivity fell for very small lesions (periapical index score 1 [42]; CBCTPAVI indices 1 and 2 [45]) and for two-dimensional slice-based analysis of small lesions [32,33]; false-positive findings were more frequent in root-filled teeth and upper second molars [41] and occurred at implant sites, near the maxillary sinus, and at the mental foramen, where artifacts and anatomical structures mimic radiolucencies [42]; and performance declined markedly in postoperative scans of root-filled molars [41]. These edge cases are precisely the situations in which a clinician is most likely to seek support, and they carry asymmetric consequences: a missed small lesion delays treatment, whereas a false-positive finding in a root-filled tooth may prompt unnecessary retreatment, apical surgery, or referral. Automation bias, the tendency to accept incorrect AI suggestions, was not examined in any included study; the one study that stratified readers by experience found that AI assistance benefited junior dentists more than senior endodontists and that the most experienced dentist performed slightly worse with assistance [50]. The operating threshold of commercial platforms is fixed by the vendor (50% in one study [41]) and cannot be adjusted to the clinical context, and several studies reported only lesion-enriched samples in which the false-positive burden of routine screening cannot be estimated.

Translation into practice would require evidence that is currently absent. Prospective reader studies that compare clinicians with and without AI assistance under a common protocol, with a reference standard established by calibrated experts blinded to the AI output and, where feasible, supplemented by histopathology or follow-up, are needed to establish whether AI changes diagnostic decisions; diagnostic randomized trials or before-after studies with patient-relevant outcomes (treatment decisions, retreatment rates, healing, and adverse events) and economic evaluations are needed to establish clinical utility and cost-effectiveness [56,57]. Implementation barriers include regulatory approval as a medical device, which requires evidence of safety and performance across populations and imaging devices and provisions for human oversight and post-market surveillance; integration into picture archiving and reporting workflows without adding reading time; generalizability across CBCT manufacturers, fields of view, voxel sizes, and reconstruction protocols, which were rarely reported and never systematically varied in the included studies; medico-legal responsibility for AI-flagged findings; and the cost of commercial platforms in settings where access to specialist interpretation is already limited. Potential benefits, such as supporting referral decisions by general practitioners or reducing interpretation variability, therefore remain plausible but undemonstrated.

Future studies should follow AI-specific reporting standards [61,62]; report complete 2 × 2 data at the tooth or root level, with the number of reference-positive and reference-negative units in the test set; use reference standards established by at least two calibrated experts in consensus and blinded to AI output, supplemented by histopathology or follow-up where feasible; validate models externally across centers, devices, and acquisition protocols; stratify performance by lesion size, jaw, tooth type, and root-filling status; and evaluate AI prospectively in reader studies with and without AI assistance, with outcomes that include clinical decision-making, patient outcomes, cost-effectiveness, and interpretability.

## 5. Conclusions

AI models showed promising performance for detecting and classifying periapical lesions on CBCT under study conditions, with summary sensitivity and specificity of 88% and 85% in the bivariate synthesis and univariate pooled estimates of approximately 84–91% for sensitivity, specificity, accuracy, and F1-score, whereas lesion segmentation achieved moderate overlap with reference annotations (DSC 0.70). The certainty of the evidence is very low, owing to considerable heterogeneity, wide prediction intervals, predominantly retrospective and internally validated designs, lesion-enriched samples, and incompletely reported reference standards, and performance falls for very small lesions and in root-filled teeth. Current evidence therefore does not establish equivalence to experienced clinicians or benefits for clinical decision-making and patient outcomes. Prospective, multicenter, externally validated studies using standardized reference standards and complete reporting of 2 × 2 data are required before AI can be recommended for routine endodontic diagnosis.

## Figures and Tables

**Table 1:** QUADAS-2 criteria applied to AI studies of periapical lesion assessment on CBCT.

Domain	Low Risk	High Risk	Unclear
Patient selection	Consecutive or random sample of scans; case-control design avoided; no inappropriate exclusions	Selective or curated sampling, case-control design, or inappropriate exclusions likely to bias performance	Sampling method insufficiently reported
Index test	Performance evaluated on data independent of training and tuning; threshold prespecified; output obtained without knowledge of the reference standard	Evaluation on training or tuning data, threshold selected post hoc, or output interpreted with knowledge of the reference standard	Data partitioning, threshold, or blinding insufficiently reported
Reference standard	Likely to classify the target condition correctly (e.g., calibrated experts in consensus, or histopathology) and established without knowledge of AI output	Unlikely to classify the target condition correctly, or established with knowledge of AI output	Procedure, reader expertise, or blinding insufficiently reported
Flow and timing	All units received the same reference standard and were included in the analysis	Partial or differential verification, or exclusions from the analysis	Flow insufficiently reported

AI, artificial intelligence; CBCT, cone-beam computed tomography; QUADAS-2, Quality Assessment of Diagnostic Accuracy Studies-2.

**Table 2:** Characteristics of the included studies.

Study	Design	Country	Sample Size	Sample Characteristics	AI Model (Type)	AI Task
Aboelmaaty et al., 2024 [43]	Retrospective observational imaging study	Egypt	240 CBCT scans	Patients with posterior maxillary periapical lesions and controls without odontogenic infection; teeth within 2 mm of the sinus floor	eVol DXS v1.0.1.0 (commercial)	PSAA localization and sinus assessment; lesion detection not evaluated
Akalin and Özkan, 2025 [32]	Model development and comparative validation	Turkey	2000 image slices (1000 sagittal-coronal pairs)	Coronal and sagittal slices of teeth with and without lesions (public dataset of Calazans et al.)	VGG16 transfer learning (in-house)	Binary lesion classification
Allihaibi et al., 2025 [41]	Retrospective diagnostic accuracy study	United Kingdom	134 molars	Preoperative and postoperative scans of root-filled and non-root-filled molars	Diagnocat (commercial)	Lesion detection
Arı et al., 2026 [44]	Model development and validation	Turkey	300 images	CBCT images labelled for 15 dental and jaw conditions	Mask R-CNN with ResNet-101 (in-house)	Multiclass detection
Boubaris et al., 2024 [45]	Validation against semi-automated method	Australia	500 roots	Roots graded with the CBCT periapical volume index (CBCTPAVI)	Diagnocat (commercial) vs. Mimics Research semi-automated segmentation	Lesion-volume grading
Calazans et al., 2022 [33]	Model development and validation	Brazil	1000 sagittal-coronal section pairs from 885 CBCT examinations	Sagittal and coronal sections of healthy teeth and teeth with endodontic lesions	Siamese network with VGG16 and DenseNet-121 backbones (in-house)	Binary lesion classification
Chau et al., 2025 [46]	Model development and comparative validation	China	185 CBCT scans	Scans with confirmed periapical lesions	CBCT-SAM with/without PPR module; compared with modified U-Net and PAL-Net (in-house)	Segmentation
Chen et al., 2024 [47]	Model development and validation	United States	138 CBCT volumes	Volumes labelled as lesion, restorative material, bone, tooth, and background	U-Net and Swin-UNETR variants (in-house)	Multiclass segmentation; lesion detection
Ezhov et al., 2021 [48]	Model development with clinical reader evaluation	Russia	1346 training scans; separate test set of 300 scans; 30 scans read by 24 dentists	Scans with dental anatomy and pathology; dentists with and without AI assistance	Diagnocat (commercial)	Multi-condition detection; AI-assisted reading
Flores et al., 2009 [49]	Computer-aided diagnosis development and validation	United States	17 CBCT scans	Scans of patients with periapical lesions	Graph-based random-walk segmentation with LDA-AdaBoost classifier (in-house)	Differential classification (cyst vs. granuloma)
Fu et al., 2024 [50]	Model development with internal and external validation	China	279 CBCT scans	Patients with apical periodontitis; multicenter datasets	PAL-Net, 3D CNN (in-house)	Detection and segmentation
Hadzic et al., 2024 [42]	Diagnostic accuracy validation study	Austria	195 CBCT scans	Adult maxillary and mandibular scans; lesions scored with a periapical index	Previously developed CNN (in-house)	Lesion detection
Huang et al., 2024 [51]	Model development and validation	United States	20 CBCT volumes (100 slices; final training set, 26 slices)	Clinician-segmented volumes with five tissue/lesion classes	Bayesian U-Net with BALD and maximum-entropy active learning (in-house)	Segmentation; lesion detection
Lee et al., 2025 [52]	Model development and validation	United States	145 CBCT volumes	Scans with small lesions, bone, teeth, and restorative materials	OAK-SSL semi-supervised framework (in-house)	Segmentation; lesion detection
Naidu et al., 2025 [36]	Model development and validation	India	500 CBCT scans	Scans with periapical cysts, abscesses, and granulomas	ML and DL classifiers (in-house)	Lesion classification
Orhan et al., 2020 [53]	Validation study	Turkey	153 lesions in 109 patients	Maxillary and mandibular periapical lesions	Diagnocat deep CNN (commercial)	Detection, localization, and volumetric measurement
Setzer et al., 2020 [54]	Model development and validation	United States	20 CBCT volumes (61 roots)	Limited field-of-view scans of roots with and without lesions	U-Net-based DL algorithm (in-house)	Detection and segmentation
Zhao et al., 2024 [55]	Model development and validation	China	600 images from 120 subjects (284 teeth)	Normal tissue, CBCTPAI 1-2, CBCTPAI 3-5, and young permanent teeth	PAINet; compared with CNN- and transformer-based models (in-house)	Lesion grading (four classes)

3D, three-dimensional; AI, artificial intelligence; BALD, Bayesian active learning by disagreement; CBCT, cone-beam computed tomography; CBCTPAI, CBCT periapical index; CNN, convolutional neural network; DL, deep learning; LDA, linear discriminant analysis; ML, machine learning; OAK-SSL, oral-anatomical knowledge-informed semi-supervised learning; PPR, prompt/prediction refinement; PSAA, posterior superior alveolar artery; SAM, Segment Anything Model.

**Table 3:** Reference standard, validation strategy, unit of analysis, and outcomes of the included studies.

Study	Reference Standard	Training/Validation/Test Split	Validation	Unit of Analysis	Outcome Measures	Key Findings
Aboelmaaty et al., 2024 [43]	Two independent experienced radiologists, calibrated (κ = 0.92)	Not applicable (commercial software)	None reported for the AI output	Scan	PSAA localization; sinus mucosal thickening	PSAA identified in 100% of scans; mucosal thickening more frequent in the lesion group when the PSAA lay beneath the sinus membrane (62.5% vs. 8.6%)
Akalin and Özkan, 2025 [32]	Labels of the public UFPE dataset (one oral and maxillofacial radiologist)	80% training, 20% test	Internal (hold-out)	Image slice	Precision, recall, F1-score	Final regularized VGG16: accuracy 58.6%, recall 58.0%, specificity 63.0%, precision 90.9%, F1-score 70.8% (unregularized baseline: recall 72%, F1-score 52%)
Allihaibi et al., 2025 [41]	Two experienced endodontists, independent, blinded to clinical information and AI output (intra-examiner κ = 0.93; inter-examiner κ = 0.74)	Not applicable (commercial software)	Independent clinical evaluation	Tooth and root	Sensitivity, specificity, accuracy, PPV, NPV, F1-score, AUC	Preoperative, tooth level (65 PARL-positive, 69 PARL-negative teeth): sensitivity 93.8%, specificity 65.2%, accuracy 79.1%, F1-score 81.3%, AUC 0.76; root level (138/189): 86.2%, 79.9%, 82.6%, 80.7%, AUC 0.79
Arı et al., 2026 [44]	NR	NR	NR	Image	Condition-specific F1-score	F1-score 0.87 for periapical lesions
Boubaris et al., 2024 [45]	Semi-automated CBCTPAVI assessment (Mimics Research); operator NR	Not applicable (commercial software)	Independent evaluation	Root	Accuracy, precision, recall, F1-score, agreement	84.4% exact CBCTPAVI agreement; no lesion scored 1 or 2 by AI; dichotomized lesion presence (408 lesions, 92 lesion-free roots): sensitivity 92.6%, specificity 96.7%, accuracy 93.4%
Calazans et al., 2022 [33]	One oral and maxillofacial radiologist (10 years’ CBCT experience) assessing the whole CBCT volume	60% training, 20% validation, 20% test	Internal (hold-out)	Image section	Accuracy, recall, precision, specificity, F1-score	Complete dataset, DenseNet-121: accuracy 70.0%, recall 64.5%, specificity 76.3%, precision 75.8%, F1-score 69.7%; accuracy 79–81% when small lesions excluded
Chau et al., 2025 [46]	Manual segmentation by a trained operator, validated by a maxillofacial radiologist	NR	Internal (5-fold cross-validation)	Scan; voxel-level metrics	Segmentation accuracy, sensitivity, specificity, precision, DSC	183/185 lesions detected (98.9%); lesion DSC 0.70 ± 0.19; voxel-level sensitivity 72.4%, specificity 99.9%, precision 0.73
Chen et al., 2024 [47]	Expert-clinician annotation	103 training, 10 validation, 25 test volumes (time-based split)	Internal (hold-out)	Volume; voxel-level (DSC)	DSC; lesion detection sensitivity and specificity	Swin-UNETR-PRETRAIN-103: lesion DSC 0.85; per-root sensitivity 1.00 (42/42), specificity 0.92 (140 lesion-free roots)
Ezhov et al., 2021 [48]	Dentomaxillofacial radiologists	Training, 1346 scans; test, 300 scans; reader study, 30 scans	Held-out test set plus reader study (24 dentists, with and without AI)	Tooth	Sensitivity and specificity of AI and of AI-assisted vs. unaided readers	Standalone AI, periapical lesion: sensitivity 83.8%, specificity 99.5% (cross-condition aggregate 92.4%/99.0%); reader sensitivity 85.4% with vs. 76.7% without AI
Flores et al., 2009 [49]	Endodontic experts’ CBCT diagnosis; histopathology by an oral pathologist	Leave-one-out cross-validation	Internal (cross-validation)	Scan	Classification accuracy	Accuracy 94.1% against expert CBCT diagnosis and 88.2% against biopsy
Fu et al., 2024 [50]	Consensus diagnosis by two endodontists (third expert arbiter); manual segmentation by two endodontists, double-checked	5-fold cross-validation (2841 teeth); independent test set (324 teeth, 36 AP); three external sets (1786 teeth, 146 AP)	Internal and external (multicenter)	Scan/volume	AUC, F1-score, DSC, diagnostic time	Independent test set: sensitivity 0.97, PPV 0.76, F1-score 0.85, AUC 0.97, DSC 0.81; cross-validation AUC 0.98, DSC 0.87; external AUC 0.93–0.96, DSC 0.80–0.90
Hadzic et al., 2024 [42]	Three investigators (two senior oral surgeons with >15 years’ experience, one junior dentist)	Not applicable (previously developed model)	External	Tooth	Sensitivity, specificity by lesion size, jaw, and tooth type	Sensitivity 86.7% (260/300 lesions), specificity 84.2% (2230/2647 lesion-free teeth); sensitivity 90.4% excluding PAI-1 lesions
Huang et al., 2024 [51]	Clinician-generated segmentation labels	Training-to-testing ratio 4:1; final training set 26 volumes	Internal (hold-out)	Lesion/root (detection); slice (DSC)	DSC, lesion detection sensitivity	BALD: sensitivity 84.0% (21/25), specificity 90.3% (28/31), lesion DSC 0.505; ME: 76.0%, 96.8%, 0.501; Bayesian U-Net without AL: 32.0%, 96.8%, 0.288
Lee et al., 2025 [52]	Manual segmentation in ITK-SNAP, reviewed and refined by oral and maxillofacial radiology experts	Training, 20 labelled plus 80 unlabeled volumes; validation, 15; test, 30 (small lesions)	Internal (hold-out); single center	Volume; voxel-level	Segmentation and lesion detection metrics	OAK-SSL lesion DSC 0.647 (30 small-lesion test volumes); per-root recall 0.933, precision 0.791
Naidu et al., 2025 [36]	Two radiologists assessed the 100 test scans (interobserver agreement 0.98; *p* < 0.001)	Training, 400 scans; test, 100 scans	Internal (hold-out)	Scan	Sensitivity, specificity, accuracy, interobserver agreement	ML: 100% sensitivity, specificity and accuracy (96 scans); DL: sensitivity 91.3%, specificity 63.4%, accuracy 84.6%; all scans lesion-positive (lesion-type classification)
Orhan et al., 2020 [53]	One oral and maxillofacial radiologist (8 years’ experience) identified lesions; manual segmentation (Fujifilm Synapse 3D)	Not applicable (commercial software)	Independent evaluation	Lesion	Detection rate, precision, F1-score, volume agreement	142/153 lesions detected (92.8%); reported recall 0.89, precision 0.95, F-measure 0.93; AI and manual volumes not significantly different
Setzer et al., 2020 [54]	Three calibrated reviewers (oral and maxillofacial radiologist, endodontist, senior graduate student); disagreements resolved by joint discussion	Five-fold cross-validation	Internal (cross-validation)	Root (detection); voxel (segmentation)	Accuracy, sensitivity, specificity, PPV, NPV, DSC	Sensitivity 0.93 (27/29), specificity 0.88 (28/32), accuracy 0.90 (55/61); lesion DSC 0.52 overall and 0.67 for true lesions
Zhao et al., 2024 [55]	Two radiologists after joint training; disagreements resolved by an expert radiologist; graded with CBCTPAI	8:1:1 (per class: 120 training, 15 validation, 15 test images; ×16 augmentation)	Internal (hold-out); images of the same tooth kept in one set	Image	Accuracy, precision, recall, F1-score, AUC, Cohen’s κ	Four-class accuracy 0.933, macro-average F1-score 0.934 and AUC 0.997, κ = 0.911 (60 test images)

AUC, area under the receiver operating characteristic curve; CBCTPAVI, CBCT periapical volume index; DSC, Dice similarity coefficient; NPV, negative predictive value; NR, not reported; PPV, positive predictive value. Other abbreviations as in Table 2. PARL, periapical radiolucency; AP, apical periodontitis; AL, active learning; ME, maximum entropy; PAI, periapical index; UFPE, Federal University of Pernambuco.

**Table 4:** QUADAS-2 risk-of-bias and applicability judgements for each included study.

Study	RoB D1	RoB D2	RoB D3	RoB D4	RoB Overall	AC D1	AC D2	AC D3
Aboelmaaty et al., 2024 [43]	Unclear	Low	Unclear	Low	Unclear	High	High	High
Akalin and Özkan, 2025 [32]	Unclear	Low	Unclear	Low	Unclear	Low	Low	Low
Allihaibi et al., 2025 [41]	Unclear	Low	Low	Low	Unclear	Low	Low	Low
Arı et al., 2026 [44]	Unclear	Low	Unclear	Low	Unclear	Low	Low	Low
Boubaris et al., 2024 [45]	Unclear	Low	Low	Low	Unclear	Low	Low	Low
Calazans et al., 2022 [33]	Unclear	Low	Low	Low	Unclear	Low	Low	Low
Chau et al., 2025 [46]	Unclear	Low	Low	Low	Unclear	Low	Low	Low
Chen et al., 2024 [47]	Unclear	Low	Low	Low	Unclear	Low	Low	Low
Ezhov et al., 2021 [48]	Low	Low	Low	Low	Low	Low	High	Low
Flores et al., 2009 [49]	Unclear	Low	Unclear	Low	Unclear	High	Low	Low
Fu et al., 2024 [50]	Low	Low	Low	Low	Low	Low	Low	Low
Hadzic et al., 2024 [42]	Low	Low	Low	Low	Low	Low	Low	Low
Huang et al., 2024 [51]	Unclear	Low	Low	Low	Unclear	Low	Low	Low
Lee et al., 2025 [52]	Unclear	Low	Low	Low	Unclear	Low	Low	Low
Naidu et al., 2025 [36]	Unclear	Low	Unclear	Low	Unclear	Low	Low	Low
Orhan et al., 2020 [53]	Unclear	Low	Low	Low	Unclear	Low	Low	Low
Setzer et al., 2020 [54]	Unclear	Low	Low	Low	Unclear	Low	Low	Low
Zhao et al., 2024 [55]	Unclear	Low	Low	Low	Unclear	Low	Low	Low

AC, applicability concern; D1, patient selection; D2, index test; D3, reference standard; D4, flow and timing; QUADAS-2, Quality Assessment of Diagnostic Accuracy Studies-2; RoB, risk of bias.

**Table 5:** Summary of the meta-analyses (primary analyses: bivariate model for sensitivity and specificity; univariate random-effects models with one estimate per study for all metrics).

Metric (Model)	Studies	Pooled Estimate (95% CI)	I^2^ (Q, df, *p*)	τ^2^	95% PI	Leave-One-Study-Out Range
Sensitivity (bivariate model) a	7	88.2% (77.6–94.2)	-	0.84	51.4–98.2%	86.4–90.5%
Specificity (bivariate model) a	7	85.1% (73.4–92.1)	-	0.80	46.0–97.4%	81.8–87.8%
Sensitivity (univariate) b	14	90.9% (84.2–94.9)	94.1% (Q = 220.02, df = 13, *p* < 0.001)	1.096	47.9–99.1%	89.4–92.3%
Specificity (univariate) c	11	89.7% (83.2–93.9)	90.4% (Q = 104.58, df = 10, *p* < 0.001)	0.690	54.3–98.5%	87.4–91.4%
Accuracy d	10	86.7% (78.9–92.0)	96.2% (Q = 233.92, df = 9, *p* < 0.001)	0.669	47.0–98.0%	85.4–88.4%
F1-score e	7	83.7% (76.4–89.1)	91.1% (Q = 67.07, df = 6, *p* < 0.001)	0.334	50.8–96.2%	81.5–85.7%
Lesion DSC f	6	0.70 (0.61–0.78)	40.9% (Q = 8.46, df = 5, *p* = 0.132)	0.091	0.46–0.86	0.68–0.72

CI, confidence interval; DSC, Dice similarity coefficient; PI, prediction interval; τ^2^, between-study variance on the logit scale (restricted maximum likelihood [REML] for the bivariate model; DerSimonian–Laird for univariate models). Univariate analyses include one prespecified estimate per study; analyses of all estimates (single-level and three-level), the other sensitivity analyses, and the full leave-one-study-out results are given in Tables S5 and S6. a—studies with complete 2 × 2 data: Akalin and Özkan [32], Allihaibi et al. (tooth level) [41], Boubaris et al. [45], Chen et al. [47], Hadzic et al. [42], Huang et al. (BALD) [51], Setzer et al. [54]; correlation between logit sensitivity and specificity ρ = 0.62; diagnostic odds ratio 43. b—Akalin [32], Allihaibi (tooth level) [41], Boubaris [45], Calazans [33], Chau [46], Chen [47], Ezhov [48], Fu (independent test set) [50], Hadzic [42], Huang (BALD) [51], Lee [52], Naidu (ML) [36], Orhan [53], Setzer [54]. c—as b, without Chau, Lee, and Orhan (lesion-positive samples). d— Akalin [32], Allihaibi [41], Boubaris [45], Calazans [33], Chen [47], Hadzic [42], Huang [51], Naidu (ML) [36], Setzer [54], Zhao [55]. e—Akalin [32], Allihaibi [41], Arı [44], Calazans [33], Fu [50], Orhan [53], Zhao [55]. f—Chau [46], Chen [47], Fu (independent test set) [50], Huang (BALD) [51], Lee [52], Setzer [54]. The area under the receiver operating characteristic curve (AUC) was not pooled (Section 3.4.3).

**Table 6:** Certainty of evidence (GRADE) for the pooled outcomes.

Outcome (Model)	Studies	Pooled Estimate (95% CI)	Risk of Bias	Indirectness	Inconsistency	Imprecision	Publication Bias	Certainty
Sensitivity (bivariate)	7	88.2% (77.6–94.2)	Serious ^a^	Serious ^b^	Very serious ^c^	Not serious	Not assessed ^e^	Very low
Specificity (bivariate)	7	85.1% (73.4–92.1)	Serious ^a^	Serious ^b^	Very serious ^c^	Serious ^d^	Not assessed ^e^	Very low
Sensitivity (univariate)	14	90.9% (84.2–94.9)	Serious ^a^	Serious ^b^	Very serious ^c^	Not serious	Not assessed ^e^	Very low
Specificity (univariate)	11	89.7% (83.2–93.9)	Serious ^a^	Serious ^b^	Very serious ^c^	Not serious	Not assessed ^e^	Very low
Accuracy	10	86.7% (78.9–92.0)	Serious ^a^	Serious ^b^	Very serious ^c^	Not serious	Not assessed ^e^	Very low
F1-score	7	83.7% (76.4–89.1)	Serious ^a^	Serious ^b^	Very serious ^c^	Not serious	Not assessed ^e^	Very low
Lesion DSC	6	0.70 (0.61–0.78)	Serious ^a^	Serious ^b^	Serious ^f^	Not serious	Not assessed ^e^	Very low

^a^ Most contributing studies were rated unclear risk of bias for patient selection (Table 4). ^b^ Predominantly lesion-enriched, internally validated development datasets; units of analysis ranged from image sections to teeth, roots, lesions, and scans. ^c^ I^2^ = 90.4–96.2% (univariate models) or large between-study variances (bivariate model), with 95% prediction intervals or regions extending below 55%. ^d^ Lower 95% confidence limit below 75%. ^e^ Fewer than ten studies per analysis; not formally assessed and not downgraded, although selective publication of small development studies is plausible. ^f^ Moderate statistical heterogeneity (I^2^ = 40.9%) but a wide 95% prediction interval (0.46–0.86). CI, confidence interval; DSC, Dice similarity coefficient; GRADE, Grading of Recommendations Assessment, Development and Evaluation.

## Data Availability

The data extracted and analyzed in this study, the per-estimate dataset, and the analysis code are included in the article and its Supplementary Materials.

## Supplementary Materials

The following supporting information can be downloaded at: https://www.mdpi.com/article/10.3390/diagnostics16213606/s1, Table S1: Complete search strategies; Table S2: Full-text articles excluded, with reasons [8,16,63,64,65,66,67,68,69,70,71,72,73]; Table S3: Structured summary of study characteristics alongside the primary estimate of each metric; Table S4: Reference-standard characteristics of each included study; Table S5: Primary, all-estimates, and sensitivity analyses for each metric; Table S6: Leave-one-study-out analyses; Table S7: Per-estimate dataset with numerators, denominators, model configuration, and provenance; Text S1: Study-by-study summary of reported standalone AI performance; Figure S1: Funnel plots for sensitivity, specificity, accuracy, F1-score, and lesion DSC, one estimate per study; Table S8: PRISMA 2020 checklist; Table S9: PRISMA 2020 for Abstracts checklist; Table S10: PRISMA-DTA checklist; Table S11: PRISMA-DTA for Abstracts checklist [74]; analysis code (Python and R).

## Abbreviations

The following abbreviations are used in this manuscript: AI, artificial intelligence; AUC, area under the receiver operating characteristic curve; BALD, Bayesian active learning by disagreement; CBCT, cone-beam computed tomography; CBCTPAI, CBCT periapical index; CBCTPAVI, CBCT periapical volume index; CI, confidence interval; CNN, convolutional neural network; DL, deep learning; DSC, Dice similarity coefficient; GRADE, Grading of Recommendations Assessment, Development and Evaluation; HSROC, hierarchical summary receiver operating characteristic; ML, machine learning; NR, not reported; PI, prediction interval; PIRD, Population, Index test, Reference standard, Diagnosis of interest; PPV, positive predictive value; PRISMA, Preferred Reporting Items for Systematic Reviews and Meta-Analyses; PRISMA-DTA, PRISMA extension for diagnostic test accuracy studies; QUADAS-2, Quality Assessment of Diagnostic Accuracy Studies-2; REML, restricted maximum likelihood; SROC, summary receiver operating characteristic; SWiM, Synthesis Without Meta-analysis.
