# Supplementary material for: Diagnostic Performance of Artificial Intelligence for the Detection and Segmentation of Periapical Lesions on Cone-Beam Computed Tomography: A Systematic Review and Meta-Analysis
Source: Diagnostics (Basel). 2026 Nov 1;16(21):3606. doi: 10.3390/diagnostics16213606 (PMC13761569; doi:10.3390/diagnostics16213606)
Supplement: Supplementary file 1 [file diagnostics-16-03606-s001.zip › diagnostics-4639763-supplementary.pdf]

Supplementary Materials

Diagnostic Performance of Artificial Intelligence for the Detection and Segmentation of Periapical Lesions on Cone-Beam Computed Tomography: A Systematic Review and Meta-Analysis

Mey A. Al-Habib and Mohammed Howait

Table S1. Complete search strategies. All sources were last searched on 12 December 2025.

Source	Field / step	Search string	Records
PubMed	#1	"Artificial intelligence"[All Fields] OR "algorithm s"[All Fields] OR "algorithmic"[All Fields] OR "algorithmically"[All Fields] OR "algorithmics"[All Fields] OR "algorithmization"[All Fields] OR "algorithms"[MeSH Terms] OR "algorithms"[All Fields] OR "algorithm"[All Fields] OR "Artificial General Intelligence"[All Fields] OR "Artificial Narrow Intelligence"[All Fields] OR "Artificial Superintelligence"[All Fields] OR "Fuzzy Logic"[All Fields] OR "Neural Networks"[All Fields] OR "Bayesian Networks"[All Fields] OR "Explainable AI"[All Fields] OR "Probabilistic Models"[All Fields] OR "Machine Learning"[All Fields] OR "Natural Language Processing"[All Fields] OR "Pattern Recognition"[All Fields] OR "Reinforcement Learning"[All Fields] OR "Data Mining"[All Fields] OR "Generative Adversarial Networks"[All Fields] OR "Probabilistic Models"[All Fields] OR "Bayesian Networks"[All Fields]	1049393
PubMed	#2	"periapical lesion"[All Fields] OR "apical lesion"[All Fields] OR "periradicular lesion"[All Fields] OR "periapical pathology"[All Fields] OR "periapical disease"[All Fields] OR "apical periodontitis"[All Fields] OR "periapical periodontitis"[All Fields] OR "periapical radiolucency"[All Fields] OR "periapical radiolucent lesion"[All Fields] OR "apical radiolucency"[All Fields] OR "periapical granuloma"[All Fields] OR "apical granuloma"[All Fields] OR "periapical cyst"[All Fields] OR "radicular cyst"[All Fields] OR "apical cyst"[All Fields] OR "periapical abscess"[All Fields] OR "apical abscess"[All Fields] OR "periapical inflammation"[All Fields] OR "apical inflammation"[All Fields]	10878
PubMed	#3	"Cone-Beam Computed Tomography"[All Fields] OR "CBCT"[All Fields] OR "Cone-Beam"[All Fields] OR "Volumetric CT"[All Fields] OR "Digital Volume Tomography"[All Fields] OR "Volumetric Tomography"[All Fields] OR "3D Dental Imaging"[All Fields]	30664
PubMed	#4	("Artificial intelligence"[All Fields] OR ("algorithm s"[All Fields] OR "algorithmic"[All Fields] OR "algorithmically"[All Fields] OR "algorithmics"[All Fields] OR "algorithmization"[All Fields] OR "algorithms"[MeSH Terms] OR "algorithms"[All Fields] OR "algorithm"[All Fields]) OR "Artificial General Intelligence"[All Fields] OR "Artificial Narrow Intelligence"[All Fields] OR "Artificial Superintelligence"[All Fields] OR "Fuzzy Logic"[All Fields] OR "Neural Networks"[All Fields] OR "Bayesian Networks"[All Fields] OR "Explainable AI"[All Fields] OR "Probabilistic Models"[All Fields] OR "Machine Learning"[All Fields] OR "Natural Language Processing"[All Fields] OR "Pattern Recognition"[All Fields] OR "Reinforcement Learning"[All Fields] OR "Data Mining"[All Fields] OR "Generative Adversarial Networks"[All Fields] OR "Probabilistic Models"[All Fields] OR "Bayesian Networks"[All Fields]) AND ("periapical lesion"[All Fields] OR "apical lesion"[All Fields] OR "periradicular lesion"[All Fields] OR "periapical pathology"[All Fields] OR "periapical disease"[All Fields] OR "apical periodontitis"[All Fields] OR "periapical periodontitis"[All Fields] OR "periapical radiolucency"[All Fields] OR "periapical radiolucent lesion"[All Fields] OR "apical radiolucency"[All Fields] OR "periapical granuloma"[All Fields] OR "apical granuloma"[All Fields] OR "periapical cyst"[All Fields] OR "radicular cyst"[All Fields] OR "apical cyst"[All Fields] OR "periapical abscess"[All Fields] OR "apical abscess"[All Fields] OR "periapical inflammation"[All Fields] OR "apical inflammation"[All Fields]) AND ("Cone-Beam Computed Tomography"[All Fields] OR "CBCT"[All Fields] OR "Cone-Beam"[All Fields] OR "Volumetric CT"[All Fields] OR "Digital Volume Tomography"[All Fields] OR "Volumetric Tomography"[All Fields] OR "3D Dental Imaging"[All Fields])	37
Cochrane Library	Title, abstract, keywords (Trials)	("Artificial intelligence" OR Algorithm OR "Artificial General Intelligence" OR "Artificial Narrow Intelligence" OR "Artificial Superintelligence" OR "Fuzzy Logic" OR "Neural Networks" OR "Bayesian Networks" OR "Explainable AI" OR "Probabilistic Models" OR "Machine Learning" OR "Natural Language Processing" OR "Pattern Recognition" OR "Reinforcement Learning" OR "Data Mining" OR "Generative Adversarial Networks" OR "Probabilistic Models" OR "Bayesian Networks") AND ("periapical lesion" OR "apical lesion" OR "periradicular lesion" OR "periapical pathology" OR "periapical disease" OR "apical periodontitis" OR "periapical periodontitis" OR "periapical radiolucency" OR "periapical radiolucent lesion" OR "apical radiolucency" OR "periapical granuloma" OR "apical granuloma" OR "periapical cyst" OR "radicular cyst" OR "apical cyst" OR "periapical abscess" OR "apical abscess" OR "periapical inflammation" OR "apical inflammation")	10

Source	Field / step	Search string	Records
Web of Science (Core Collection)	Topic (title, abstract, author keywords)	("Artificial intelligence" OR Algorithm OR "Artificial General Intelligence" OR "Artificial Narrow Intelligence" OR "Artificial Superintelligence" OR "Fuzzy Logic" OR "Neural Networks" OR "Bayesian Networks" OR "Explainable AI" OR "Probabilistic Models" OR "Machine Learning" OR "Natural Language Processing" OR "Pattern Recognition" OR "Reinforcement Learning" OR "Data Mining" OR "Generative Adversarial Networks") AND ("periapical lesion" OR "apical lesion" OR "periradicular lesion" OR "periapical pathology" OR "periapical disease" OR "apical periodontitis" OR "periapical periodontitis" OR "periapical radiolucency" OR "periapical radiolucent lesion" OR "apical radiolucency" OR "periapical granuloma" OR "apical granuloma" OR "periapical cyst" OR "radicular cyst" OR "apical cyst" OR "periapical abscess" OR "apical abscess" OR "periapical inflammation" OR "apical inflammation") AND ("Cone-Beam Computed Tomography" OR CBCT OR "Cone-Beam" OR "Volumetric CT" OR "Digital Volume Tomography" OR "Volumetric Tomography" OR "3D Dental Imaging")	71
Scopus	TITLE-ABS-KEY	(TITLE-ABS-KEY (("Artificial intelligence" OR Algorithm OR "Artificial General Intelligence" OR "Artificial Narrow Intelligence" OR "Artificial Superintelligence" OR "Fuzzy Logic" OR "Neural Networks" OR "Bayesian Networks" OR "Explainable AI" OR "Probabilistic Models" OR "Machine Learning" OR "Natural Language Processing" OR "Pattern Recognition" OR "Reinforcement Learning" OR "Data Mining" OR "Generative Adversarial Networks" OR "Probabilistic Models" OR "Bayesian Networks")) AND TITLE-ABS-KEY (("periapical lesion" OR "apical lesion" OR "periradicular lesion" OR "periapical pathology" OR "periapical disease" OR "apical periodontitis" OR "periapical periodontitis" OR "periapical radiolucency" OR "periapical radiolucent lesion" OR "apical radiolucency" OR "periapical granuloma" OR "apical granuloma" OR "periapical cyst" OR "radicular cyst" OR "apical cyst" OR "periapical abscess" OR "apical abscess" OR "periapical inflammation" OR "apical inflammation")) AND TITLE-ABS-KEY (("Cone-Beam Computed Tomography" OR CBCT OR " Cone-Beam" OR "Volumetric CT" OR "Digital Volume Tomography" OR "Volumetric Tomography" OR "3D Dental Imaging")))	74
Google Scholar	All fields; sorted by relevance; all records screened	("Artificial intelligence" OR Algorithm OR "Artificial General Intelligence" OR "Artificial Narrow Intelligence" OR "Artificial Superintelligence" OR "Fuzzy Logic" OR "Neural Networks" OR "Bayesian Networks" OR "Explainable AI" OR "Probabilistic Models" OR "Machine Learning" OR "Natural Language Processing" OR "Pattern Recognition" OR "Reinforcement Learning" OR "Data Mining" OR "Generative Adversarial Networks" OR "Probabilistic Models" OR "Bayesian Networks") AND ("periapical lesion" OR "apical lesion" OR "periradicular lesion" OR "periapical pathology" OR "periapical disease" OR "apical periodontitis" OR "periapical periodontitis" OR "periapical radiolucency" OR "periapical radiolucent lesion" OR "apical radiolucency" OR "periapical granuloma" OR "apical granuloma" OR "periapical cyst" OR "radicular cyst" OR "apical cyst" OR "periapical abscess" OR "apical abscess" OR "periapical inflammation" OR "apical inflammation") AND ("Cone-Beam Computed Tomography" OR CBCT OR" Cone-Beam" OR "Volumetric CT" OR "Digital Volume Tomography" OR "Volumetric Tomography" OR "3D Dental Imaging")	49
ClinicalTrials.gov; WHO ICTRP	All fields	("artificial intelligence" OR "machine learning" OR "neural networks" OR algorithm) AND ("periapical lesion" OR "apical periodontitis" OR "periapical periodontitis" OR "periapical cyst" OR "radicular cyst" OR "periapical granuloma" OR "periapical abscess") AND ("cone-beam computed tomography" OR CBCT OR "cone-beam")	0
Websites and organisations	American Association of Endodontists; European Society of Endodontology; American Academy of Oral and Maxillofacial Radiology	"Artificial intelligence" OR "deep learning", combined with "periapical lesion" OR "apical periodontitis", and "CBCT" OR "cone-beam computed tomography"	4

ICTRP, International Clinical Trials Registry Platform; WHO, World Health Organization. PubMed steps #1-#3 show the AI, periapical-lesion, and CBCT blocks as translated by automatic term mapping; #4 combines them. No date, language, or publication-type limits were applied; the Cochrane Library search was restricted to the Trials (CENTRAL) collection.

Table S2. Full-text articles excluded, with reasons (n = 13).

No.	Reference	Reason for exclusion
1	Ekert, T.; Krois, J.; Meinhold, L.; et al. Deep learning for the radiographic detection of apical lesions. *J. Endod.* **2019**, *45*, 917-922.e5.	Did not use CBCT (panoramic radiographs)
2	Endres, M.G.; Hillen, F.; Salloumis, M.; et al. Development of a deep learning algorithm for periapical disease detection in dental radiographs. *Diagnostics* **2020**, *10*, 430.	Did not use CBCT (panoramic radiographs)
3	Li, C.W.; Lin, S.Y.; Chou, H.S.; et al. Detection of dental apical lesions using CNNs on periapical radiograph. *Sensors* **2021**, *21*, 7049.	Did not use CBCT (periapical radiographs)
4	Bayrakdar, I.S.; Orhan, K.; Çelik, Ö.; et al. A U-Net approach to apical lesion segmentation on panoramic radiographs. *Biomed Res. Int.* **2022**, *2022*, 7035367.	Did not use CBCT (panoramic radiographs)
5	Hamdan, M.H.; Tuzova, L.; Mol, A.; Tawil, P.Z.; Tuzoff, D.; Tyndall, D.A. The effect of a deep-learning tool on dentists' performances in detecting apical radiolucencies on periapical radiographs. *Dentomaxillofac. Radiol.* **2022**, *51*, 20220122.	Did not use CBCT (periapical radiographs)
6	Ba-Hattab, R.; et al. Detection of periapical lesions on panoramic radiographs using deep learning. *Appl. Sci.* **2023**, *13*, 1516.	Did not use CBCT (panoramic radiographs)
7	Simon, J.H.S.; Enciso, R.; Malfaz, J.M.; et al. Differential diagnosis of large periapical lesions using cone-beam computed tomography measurements and biopsy. *J. Endod.* **2006**, *32*, 833-837.	Did not evaluate an AI model
8	Estrela, C.; Bueno, M.R.; Leles, C.R.; Azevedo, B.; Azevedo, J.R. Accuracy of cone beam computed tomography and panoramic and periapical radiography for detection of apical periodontitis. *J. Endod.* **2008**, *34*, 273-279.	Did not evaluate an AI model
9	Patel, S.; Dawood, A.; Mannocci, F.; Wilson, R.; Pitt Ford, T. Detection of periapical bone defects in human jaws using cone beam computed tomography and intraoral radiography. *Int. Endod. J.* **2009**, *42*, 507-515.	Did not evaluate an AI model
10	Ahlowalia, M.S.; Patel, S.; Anwar, H.M.S.; et al. Accuracy of CBCT for volumetric measurement of simulated periapical lesions. *Int. Endod. J.* **2013**, *46*, 538-546.	Did not evaluate an AI model
11	Aminoshariae, A.; Kulild, J.; Nagendrababu, V. Artificial intelligence in endodontics: current applications and future directions. *J. Endod.* **2021**, *47*, 1352-1357.	Review article
12	Sadr, S.; Mohammad-Rahimi, H.; Motamedian, S.R.; et al. Deep learning for detection of periapical radiolucent lesions: a systematic review and meta-analysis of diagnostic test accuracy. *J. Endod.* **2023**, *49*, 248-261.e3.	Review article
13	Pul, U.; Schwendicke, F. Artificial intelligence for detecting periapical radiolucencies: a systematic review and meta-analysis. *J. Dent.* **2024**, *147*, 105104.	Review article

AI, artificial intelligence; CBCT, cone-beam computed tomography.

Table S3. Structured summary of study characteristics alongside the primary estimate of each metric.

Study	AI task	Architecture	Source	Input data	Training set	Unit of analysis	Validation strategy	Reference standard	Test set (positives)	Overall RoB	Sens. (%)	Spec. (%)	Acc. (%)	F1 (%)	DSC	AUC
Akalin and Özkan, 2025 [32]	Classification	CNN (VGG16 + regularization)	In-house	2D coronal/sagittal slices	≈1600 slices (80%)	Image slice	Internal hold-out (20%)	Public dataset labels (single radiologist)	399 slices (345 lesion, 54 healthy)	Unclear	58.0	63.0	58.6	70.8	-	-
Allihaibi et al., 2025 [41]	Detection	Commercial CNN (Diagnocat)	Commercial	3D volumes (commercial pipeline)	Not applicable	Tooth (root)	Independent clinical evaluation	2 endodontists, blinded	134 teeth (65 PARL+); 327 roots (138 PARL+)	Unclear	93.8	65.2	79.1	81.3	-	0.76
Arı et al., 2025 [44]	Multiclass detection	Mask R-CNN (ResNet-101)	In-house	2D images	NR	Image	NR	NR	NR (300 images in total)	Unclear	-	-	-	87.0	-	-
Boubaris et al., 2024 [45]	Grading (dichotomized)	Commercial CNN (Diagnocat)	Commercial	3D volumes (commercial pipeline)	Not applicable	Root	Independent evaluation	Semi-automated segmentation	500 roots (408 lesion)	Unclear	92.6	96.7	93.4	-	-	-
Calazans et al., 2022 [33]	Classification	Siamese CNN (DenseNet-121)	In-house	2D sagittal + coronal section pairs	600 pairs (60%)	Image pair	Internal hold-out (20%)	1 radiologist	≈200 pairs (≈109 lesion)	Unclear	64.5	76.3	70.0	69.7	-	-
Chau et al., 2025 [46]	Detection + segmentation	Foundation model (CBCT-SAM)	In-house	3D volumes	5-fold CV on 185 scans	Lesion/scan; voxel	Internal 5-fold CV	Operator + radiologist validation	185 lesion-positive scans	Unclear	98.9	-	-	-	0.70	-
Chen et al., 2024 [47]	Segmentation + detection	Transformer (Swin-UNETR, pretrained)	In-house	3D volumes	103 volumes (also 20/40/60)	Root; volume	Internal hold-out (time-based)	3 clinicians, consensus	25 volumes (42 lesion roots, 140 lesion-free)	Unclear	100.0	92.1	94.0	-	0.85	-
Ezhov et al., 2021 [48]	Multi-condition detection	Commercial CNN (Diagnocat)	Commercial	3D volumes	1346 scans	Tooth	Internal hold-out (300 scans)	≥3 radiologists, majority vote	≥300 positive and ≥300 negative teeth	Low	83.8	99.5	-	-	-	-
Fu et al., 2024 [50]	Detection + segmentation	3D CNN (PAL-Net)	In-house	3D volumes	2841 teeth, 178 patients (5-fold CV)	Tooth; lesion	Independent internal test set; 3 external centres	2 endodontists + arbiter, consensus	324 teeth (36 AP) test; 1786 teeth (146 AP) external	Low	97.2	96.2	-	85.0	0.81	0.97
Hadzic et al., 2024 [42]	Detection	CNN (SpatialConfiguration-Net based)	In-house	3D volumes (pre-trained model)	Not applicable (model of Kirnbauer et al.)	Tooth	External (new clinical dataset, same centre)	3 investigators, consensus	2947 teeth (300 lesion)	Low	86.7	84.2	84.5	-	-	-
Huang et al., 2024 [51]	Segmentation + detection	Bayesian U-Net + active learning	In-house	2D slices (5 per volume)	80-slice pool; 26 slices after active learning	Lesion/root; slice	Internal hold-out (4:1)	3 clinicians, consensus	20 slices (25 lesions, 56 roots)	Unclear	84.0	90.3	87.5	-	0.51	-
Lee et al., 2025 [52]	Segmentation + detection	Semi-supervised CNN (OAK-SSL)	In-house	3D volumes	20 labelled + 80 unlabelled volumes	Root; volume	Internal hold-out	Experts (number NR)	30 volumes	Unclear	93.3	-	-	-	0.65	-
Naidu et al., 2025 [36]	Classification (lesion type)	ML and DL classifiers	In-house	NR	400 scans	Scan	Internal hold-out (100 scans)	2 radiologists	100 scans (all lesion-positive)	Unclear	100.0	100.0	100.0	-	-	-
Orhan et al., 2020 [53]	Detection	Commercial CNN (Diagnocat)	Commercial	3D volumes (commercial pipeline)	Not applicable	Lesion	Independent evaluation	1 radiologist	153 lesions (109 patients)	Unclear	92.8	-	-	93.0	-	-
Setzer et al., 2020 [54]	Detection + segmentation	U-Net	In-house	2D slices from limited-FOV volumes	5-fold CV on 20 volumes	Root; volume	Internal 5-fold CV	3 clinicians, consensus	61 roots (29 lesion); 20 volumes	Unclear	93.1	87.5	90.2	-	0.67	-
Zhao et al., 2024 [55]	Grading (4 classes)	Hybrid CNN (PAINet)	In-house	2D images	480 images (×16 augmentation)	Image	Internal hold-out (8:1:1)	2 radiologists + arbiter	60 test images (15 per class)	Unclear	-	-	93.3	93.4	-	0.997

Values are the primary estimates (one per study) used in the univariate meta-analyses; all estimates, with numerators, denominators, and sources, are listed in Table S7. Aboelmaaty et al. [43] and Flores et al. [49] are not shown because they contributed to the narrative synthesis only. Acc., accuracy; AUC, area under the receiver operating characteristic curve (not pooled); CV, cross-validation; DSC, Dice similarity coefficient; NR, not reported; PARL, periapical radiolucency; RoB, risk of bias (QUADAS-2); Sens., sensitivity; Spec., specificity.

Table S4. Reference-standard characteristics of each included study.

Study	Reference standard	Readers or annotators	Expertise	Consensus procedure	Calibration or agreement	Blinding to AI output	Sampling and test-set composition
Aboelmaaty et al., 2024 [43]	Expert CBCT interpretation (PSAA location, sinus thickening; lesion detection not evaluated)	2	Experienced radiologists	Independent reading; disagreements discussed to consensus	Calibrated; Cohen κ = 0.92	NR	Retrospective; cases with posterior maxillary lesions and controls
Akalin and Özkan, 2025 [32]	Labels of the public UFPE dataset (Calazans et al.)	1 (dataset origin)	Oral and maxillofacial radiologist, 10 years of CBCT experience (per dataset description)	Not applicable	NR	Not applicable (pre-existing labels)	Public dataset; 80/20 split
Allihaibi et al., 2025 [41]	Expert CBCT assessment of PARL presence (tooth and root level)	2	Experienced endodontists	Independent scoring; disagreements resolved	Intra-examiner κ = 0.93; inter-examiner κ = 0.74	Yes (blinded to clinical information and AI output)	Convenience sample from three prior clinical studies; molars only
Arı et al., 2025 [44]	Segmentation labelling of 15 conditions	NR (full text unavailable)	NR	NR	NR	NR	300 CBCT images; split NR
Boubaris et al., 2024 [45]	Semi-automatic lesion segmentation (Mimics Research) and CBCTPAVI scoring	NR	NR	NR	NR	NR	Archive sample; 408 lesion roots, 92 root-filled lesion-free roots
Calazans et al., 2022 [33]	Classification of teeth as healthy or with endodontic lesion on the whole CBCT volume	1	Oral and maxillofacial radiologist, 10 years of CBCT experience	Not applicable	NR	Not applicable (labels preceded model development)	Curated image pairs; 454 healthy, 546 lesion
Chau et al., 2025 [46]	Manual lesion segmentation	1 operator + 1 validator	Trained operator (10-h training); expert maxillofacial radiologist validated labels	Validation of all labels by the radiologist	Training/validation on 20 scans	NR	Lesion-positive scans only (185 lesions)
Chen et al., 2024 [47]	Expert voxel-wise segmentation (five labels)	3	General dentist (2 years of segmentation experience), endodontist and oral radiologist (>20 years each) supervising	Disagreements resolved by joint discussion	Protocol from previous studies	NR	Time-based split; 25 test volumes (42 lesion roots, 140 lesion-free roots)
Ezhov et al., 2021 [48]	Tooth-level annotation of 30+ conditions by radiologists	≥3 per case (majority vote)	Experienced dentomaxillofacial radiologists; coordinator with 18 years of experience	Majority (≥2 of 3) consensus, final review by coordinator	Annotator training and evaluation before annotation	Ground truth established before AI evaluation	Consecutive scans from three clinics; test set sampled to ≥300 positives and negatives per condition
Flores et al., 2009 [49]	Endodontic experts' CBCT diagnosis; histopathology	NR	Endodontic experts; oral pathologist (biopsy)	NR	NR	NR	17 lesions with biopsy
Fu et al., 2024 [50]	Consensus clinical/radiographic diagnosis of apical periodontitis; manual 3D segmentation	2 (+1 arbiter)	Endodontists; third expert for disagreements	Joint discussion until consensus; third expert if needed	Trained endodontists	NR	Internal 5-fold CV, independent internal test set and three external centres
Hadzic et al., 2024 [42]	Tooth-level lesion presence (expert consensus)	3	Two senior oral surgeons (>15 years) and one junior dentist	Independent initial round, then consensus	NR	Ground truth established before the software was run; software output assessed afterwards in consensus by two of the three investigators	Clinically representative consecutive dataset (195 scans, 2947 present teeth)
Huang et al., 2024 [51]	Clinician voxel-wise segmentation (five labels)	3	Two senior graduate students (endodontic honours programme) supervised by an endodontist	Disagreements resolved by joint discussion including an oral radiologist	Calibrated and trained	NR	20 limited-FOV volumes; 20 test slices (25 lesions, 56 roots)
Lee et al., 2025 [52]	Manual segmentation (ITK-SNAP) reviewed and refined by experts	NR	Dental and oral radiology experts	Review and refinement	NR	NR	145 volumes, all with at least one lesion; 30 test volumes with small lesions
Naidu et al., 2025 [36]	Radiologist interpretation of CBCT (lesion type)	1 (training set); 2 (test set)	Trained radiologist; two radiologists for the 100 test scans	Interobserver agreement reported (κ = 0.98)	NR	NR	All scans with previously detected periapical pathology (lesion-positive)

Study	Reference standard	Readers or annotators	Expertise	Consensus procedure	Calibration or agreement	Blinding to AI output	Sampling and test-set composition
Orhan et al., 2020 [53]	Lesion identification and manual volumetric segmentation (Fujifilm Synapse 3D)	1	Oral and maxillofacial radiologist, 8 years of experience	Not applicable	NR	NR	Lesion-positive sample (153 lesions, 109 patients)
Setzer et al., 2020 [54]	Clinician voxel-wise segmentation (five labels)	3	Oral and maxillofacial radiologist, endodontist, senior graduate student	Initial agreement 27/29 lesions; disagreements resolved by joint discussion	Calibrated and trained	NR	20 limited-FOV volumes with at least one lesion each (61 roots)
Zhao et al., 2024 [55]	CBCTPAI grading of periapical status	2 (+1 arbiter)	Two radiologists after unified training; expert radiologist for disagreements	Joint classification; expert decision on disagreement	Unified training (2 weeks)	NR	Curated four-class dataset; images of one tooth kept within one split

NR, not reported; PSAA, posterior superior alveolar artery; UFPE, Federal University of Pernambuco public dataset.

Table S5. Primary, all-estimates, and sensitivity analyses for each metric. (a) Univariate random-effects models.

Metric	Analysis	Studies	Estimates	Pooled (95% CI)	I^2 (%)	Q (df); p	τ^2	95% PI
Sensitivity	All estimates, single-level model	14	21	88.6 (82.7-92.7)	92.5	266.39 (20); p < 0.001	1.018	46.9-98.6
Sensitivity	One estimate per study (primary analysis)	14	14	90.9 (84.2-94.9)	94.1	220.02 (13); p < 0.001	1.096	47.9-99.1
Sensitivity	One estimate per study, verified test-set denominators only	10	10	92.3 (83.5-96.6)	95.3	191.68 (9); p < 0.001	1.590	35.6-99.6
Sensitivity	One estimate per study, excluding Naidu et al. (100% estimates)	13	13	90.0 (82.7-94.4)	94.3	212.27 (12); p < 0.001	1.064	45.5-99.0
Sensitivity	One estimate per study, excluding Akalin and Özkan (same dataset as Calazans et al.)	13	13	91.7 (86.7-95.0)	86.7	90.19 (12); p < 0.001	0.629	63.7-98.6
Sensitivity	One estimate per study, independent evaluations of existing models or platforms	4	4	91.3 (87.1-94.2)	66.7	9.01 (3); p = 0.029	0.123	63.6-98.4
Sensitivity	One estimate per study, model-development studies	10	10	90.3 (81.3-95.2)	92.1	114.33 (9); p < 0.001	1.028	43.2-99.1
Sensitivity	All estimates, three-level model (REML; estimates nested within studies)	14	21	89.0 (81.7-93.6)			0.597	46.3-98.7
Specificity	All estimates, single-level model	11	18	92.5 (87.9-95.4)	94.1	290.23 (17); p < 0.001	1.042	56.7-99.1
Specificity	One estimate per study (primary analysis)	11	11	89.7 (83.2-93.9)	90.4	104.58 (10); p < 0.001	0.690	54.3-98.5
Specificity	One estimate per study, verified test-set denominators only	8	8	86.5 (77.8-92.2)	87.4	55.52 (7); p < 0.001	0.546	47.5-97.9
Specificity	One estimate per study, excluding Naidu et al. (100% estimates)	10	10	88.7 (81.7-93.2)	90.8	98.17 (9); p < 0.001	0.655	52.0-98.3
Specificity	One estimate per study, excluding Akalin and Özkan (same dataset as Calazans et al.)	10	10	91.4 (85.4-95.1)	89.7	87.57 (9); p < 0.001	0.677	58.3-98.8
Specificity	One estimate per study, independent evaluations of existing models or platforms	3	3	85.1 (67.9-93.9)	92.1	25.36 (2); p < 0.001	0.658	0.0-100.0
Specificity	One estimate per study, model-development studies	8	8	92.4 (82.5-96.9)	90.9	76.77 (7); p < 0.001	1.505	32.5-99.7
Specificity	All estimates, three-level model (REML; estimates nested within studies)	11	18	91.0 (81.8-95.8)			1.207	37.8-99.4
Accuracy	All estimates, single-level model	10	12	86.0 (79.5-90.6)	95.3	234.68 (11); p < 0.001	0.537	52.5-97.1
Accuracy	One estimate per study (primary analysis)	10	10	86.7 (78.9-92.0)	96.2	233.92 (9); p < 0.001	0.669	47.0-98.0
Accuracy	One estimate per study, verified test-set denominators only	8	8	84.3 (74.4-90.8)	96.7	212.24 (7); p < 0.001	0.650	39.3-97.8
Accuracy	One estimate per study, excluding Naidu et al. (100% estimates)	9	9	85.5 (77.1-91.2)	96.5	226.84 (8); p < 0.001	0.653	43.7-97.8
Accuracy	One estimate per study, excluding Akalin and Özkan (same dataset as Calazans et al.)	9	9	88.4 (82.7-92.3)	90.5	84.01 (8); p < 0.001	0.368	62.0-97.3
Accuracy	One estimate per study, independent evaluations of existing models or platforms	3	3	86.9 (77.8-92.6)	93.3	29.98 (2); p < 0.001	0.291	0.2-100.0
Accuracy	One estimate per study, model-development studies	7	7	87.4 (75.4-94.0)	94.0	100.18 (6); p < 0.001	0.979	30.5-99.1
Accuracy	All estimates, three-level model (REML; estimates nested within studies)	10	12	85.7 (78.3-90.9)			0.594	54.9-96.7
F1-score	All estimates, single-level model	7	11	84.8 (80.0-88.7)	92.3	129.34 (10); p < 0.001	0.280	61.3-95.2
F1-score	One estimate per study (primary analysis)	7	7	83.7 (76.4-89.1)	91.1	67.07 (6); p < 0.001	0.334	50.8-96.2
F1-score	One estimate per study, verified test-set denominators only	6	6	83.0 (74.7-89.0)	90.7	53.88 (5); p < 0.001	0.335	45.8-96.6
F1-score	One estimate per study, excluding Akalin and Özkan (same dataset as Calazans et al.)	6	6	85.6 (78.3-90.7)	88.3	42.55 (5); p < 0.001	0.320	51.5-97.1
F1-score	One estimate per study, independent evaluations of existing models or platforms	2	2	88.1 (71.3-95.7)	88.0	8.35 (1); p = 0.004	0.549	NA
F1-score	One estimate per study, model-development studies	5	5	81.7 (72.5-88.3)	91.9	49.59 (4); p < 0.001	0.309	38.5-97.0

Metric	Analysis	Studies	Estimates	Pooled (95% CI)	I² (%)	Q (df); p	τ²	95% PI
F1-score	All estimates, three-level model (REML; estimates nested within studies)	7	11	84.5 (77.7-89.5)			0.135	59.6-95.3
AUC †	All estimates, single-level model	3	7	0.92 (0.85-0.96)	95.1	121.48 (6); p < 0.001	0.808	0.50-0.99
AUC †	One estimate per study (primary analysis)	3	3	0.94 (0.66-0.99)	94.9	39.50 (2); p < 0.001	2.703	0.00-1.00
AUC †	One estimate per study, verified test-set denominators only	3	3	0.94 (0.66-0.99)	94.9	39.50 (2); p < 0.001	2.703	0.00-1.00
AUC †	One estimate per study, model-development studies	2	2	0.97 (0.95-0.98)	0.0	0.95 (1); p = 0.330	0.000	NA
AUC †	All estimates, three-level model (REML; estimates nested within studies)	3	7	0.92 (0.68-0.99)			1.766	0.34-1.00
Lesion DSC	All estimates, single-level model	6	11	0.70 (0.61-0.78)	72.2	35.93 (10); p < 0.001	0.351	0.36-0.91
Lesion DSC	One estimate per study (primary analysis)	6	6	0.70 (0.61-0.78)	40.9	8.46 (5); p = 0.132	0.091	0.46-0.86
Lesion DSC	One estimate per study, verified test-set denominators only	6	6	0.70 (0.61-0.78)	40.9	8.46 (5); p = 0.132	0.091	0.46-0.86
Lesion DSC	One estimate per study, model-development studies	6	6	0.70 (0.61-0.78)	40.9	8.46 (5); p = 0.132	0.091	0.46-0.86
Lesion DSC	All estimates, three-level model (REML; estimates nested within studies)	6	11	0.70 (0.56-0.80)			0.398	0.37-0.90

Estimates are percentages except for AUC and DSC (proportions). Univariate models: DerSimonian-Laird random effects on the logit scale; three-level model: REML, with τ² showing the between-study variance component (the within-study component is reported in the text). † AUC was not pooled in the review (Section 2.9); these analyses are shown for completeness only. CI, confidence interval; PI, prediction interval; NA, not estimable (two studies).

(b) Bivariate random-effects models of sensitivity and specificity (REML).

Analysis	Studies	Sensitivity (95% CI)	Specificity (95% CI)	τ² sens.	τ² spec.	ρ	DOR	95% PI sens.	95% PI spec.
Primary: studies with exact 2×2 tables	7	88.2 (77.6-94.2)	85.1 (73.4-92.1)	0.84	0.80	0.62	43	51.4-98.2	46.0-97.4
Adding three studies with approximate 2×2 tables	10	86.7 (77.3-92.6)	89.2 (78.9-94.8)	0.87	1.41	0.45	54	48.5-97.8	41.3-99.0
Restricted to the two studies originally identified	2	90.7 (80.1-96.0)	76.5 (53.8-90.1)	0.34	0.52	-1.00	32	69.6-97.7	36.1-94.9
Exact 2×2 tables, excluding Akalin and Özkan	6	90.5 (86.4-93.5)	87.8 (77.5-93.7)	0.10	0.67	0.31	68	82.1-95.2	55.0-97.7

DOR, diagnostic odds ratio; ρ, between-study correlation of logit sensitivity and logit specificity. The 2×2 tables are given in Table S7b.

Table S6. Leave-one-study-out analyses. (a) Univariate models (one estimate per study).

Metric	Study omitted	Studies remaining	I² (%)	Pooled (95% CI)
Sensitivity	Akalin and Özkan, 2025 [32]	13	86.7	91.7 (86.7-95.0)
Sensitivity	Allihaibi et al., 2025 [41]	13	94.4	90.6 (83.4-94.9)
Sensitivity	Boubaris et al., 2024 [45]	13	93.1	90.6 (83.5-94.8)
Sensitivity	Calazans et al., 2022 [33]	13	94.2	92.3 (85.9-96.0)
Sensitivity	Chau et al., 2025 [46]	13	94.0	89.4 (81.9-94.1)
Sensitivity	Chen et al., 2024 [47]	13	94.4	90.3 (83.1-94.6)
Sensitivity	Ezhov et al., 2021 [48]	13	94.4	91.8 (84.4-95.8)
Sensitivity	Fu et al., 2024 [50]	13	94.4	90.4 (83.1-94.7)
Sensitivity	Hadzic et al., 2024 [42]	13	94.2	91.5 (84.2-95.6)
Sensitivity	Huang et al., 2024 [51]	13	94.5	91.4 (84.5-95.3)
Sensitivity	Lee et al., 2025 [52]	13	94.5	90.7 (83.6-94.9)
Sensitivity	Naidu et al., 2025 [36]	13	94.3	90.0 (82.7-94.4)
Sensitivity	Orhan et al., 2020 [53]	13	94.1	90.7 (83.5-94.9)
Sensitivity	Setzer et al., 2020 [54]	13	94.5	90.7 (83.7-94.9)
Specificity	Akalin and Özkan, 2025 [32]	10	89.7	91.4 (85.4-95.1)
Specificity	Allihaibi et al., 2025 [41]	10	89.6	91.4 (85.2-95.1)
Specificity	Boubaris et al., 2024 [45]	10	90.6	88.6 (81.3-93.3)
Specificity	Calazans et al., 2022 [33]	10	91.0	91.1 (84.2-95.1)
Specificity	Chen et al., 2024 [47]	10	90.8	89.5 (82.1-94.0)
Specificity	Ezhov et al., 2021 [48]	10	89.5	87.4 (80.2-92.3)
Specificity	Fu et al., 2024 [50]	10	88.5	88.1 (80.9-92.8)
Specificity	Hadzic et al., 2024 [42]	10	91.4	91.4 (82.2-96.0)
Specificity	Huang et al., 2024 [51]	10	91.3	89.7 (82.7-94.1)
Specificity	Naidu et al., 2025 [36]	10	90.8	88.7 (81.7-93.2)
Specificity	Setzer et al., 2020 [54]	10	91.4	90.0 (83.0-94.3)
Accuracy	Akalin and Özkan, 2025 [32]	9	90.5	88.4 (82.7-92.3)
Accuracy	Allihaibi et al., 2025 [41]	9	96.6	87.7 (79.3-93.0)
Accuracy	Boubaris et al., 2024 [45]	9	95.8	85.4 (76.7-91.2)
Accuracy	Calazans et al., 2022 [33]	9	96.3	88.4 (80.3-93.4)
Accuracy	Chen et al., 2024 [47]	9	96.3	85.5 (76.7-91.3)
Accuracy	Hadzic et al., 2024 [42]	9	95.8	87.7 (77.1-93.8)
Accuracy	Huang et al., 2024 [51]	9	96.6	86.7 (78.3-92.2)
Accuracy	Naidu et al., 2025 [36]	9	96.5	85.5 (77.1-91.2)
Accuracy	Setzer et al., 2020 [54]	9	96.5	86.4 (77.8-91.9)
Accuracy	Zhao et al., 2024 [55]	9	96.5	85.9 (77.3-91.6)
F1-score	Akalin and Özkan, 2025 [32]	6	88.3	85.6 (78.3-90.7)
F1-score	Allihaibi et al., 2025 [41]	6	92.5	84.2 (75.8-90.1)
F1-score	Arı et al., 2025 [44]	6	90.7	83.0 (74.7-89.0)
F1-score	Calazans et al., 2022 [33]	6	90.8	85.7 (78.1-91.0)
F1-score	Fu et al., 2024 [50]	6	91.5	83.6 (74.9-89.7)
F1-score	Orhan et al., 2020 [53]	6	90.1	81.5 (73.8-87.4)
F1-score	Zhao et al., 2024 [55]	6	91.7	82.2 (74.2-88.1)
AUC †	Allihaibi et al., 2025 [41]	2	0.0	0.97 (0.95-0.98)
AUC †	Fu et al., 2024 [50]	2	73.1	0.95 (0.17-1.00)
AUC †	Zhao et al., 2024 [55]	2	97.3	0.91 (0.51-0.99)
Lesion DSC	Chau et al., 2025 [46]	5	52.7	0.70 (0.57-0.81)
Lesion DSC	Chen et al., 2024 [47]	5	30.6	0.68 (0.60-0.76)
Lesion DSC	Fu et al., 2024 [50]	5	35.8	0.68 (0.59-0.76)
Lesion DSC	Huang et al., 2024 [51]	5	16.4	0.72 (0.65-0.78)
Lesion DSC	Lee et al., 2025 [52]	5	50.2	0.71 (0.61-0.80)
Lesion DSC	Setzer et al., 2020 [54]	5	52.3	0.71 (0.60-0.79)

† AUC was not pooled in the review; shown for completeness only.

(b) Bivariate model (studies with exact 2×2 tables).

**Study omitted**	**Sensitivity (95% CI)**	**Specificity (95% CI)**
Akalin and Özkan, 2025 [32]	90.5 (86.4-93.5)	87.8 (77.5-93.7)
Allihaibi et al., 2025 [41]	87.3 (76.0-93.7)	85.9 (76.4-92.0)
Boubaris et al., 2024 [45]	88.2 (74.1-95.2)	81.8 (70.0-89.6)
Chen et al., 2024 [47]	86.4 (74.9-93.1)	83.7 (69.2-92.1)
Hadzic et al., 2024 [42]	89.0 (75.6-95.5)	85.4 (70.8-93.4)
Huang et al., 2024 [51]	89.1 (76.5-95.4)	84.4 (70.5-92.5)
Setzer et al., 2020 [54]	87.6 (74.9-94.3)	84.8 (70.6-92.8)

Table S7. (a) Per-estimate dataset with numerators, denominators, model configuration, and provenance.

Metric	Study	Estimate	Unit	Test set	Model	Events	N	Value	Primary	Source and derivation
Sensitivity	Akalin and Özkan, 2025 [32]		image slice	internal hold-out (20%)	VGG16 + regularization 13	200	345	58.0	Yes	Table 3 (Reg13): TP 200, FN 145
Sensitivity	Allihaibi et al., 2025 [41]	tooth level	tooth	independent clinical evaluation	Diagnocat	61	65	93.8	Yes	Table 1/Suppl: 65 PARL-positive teeth; 93.8%
Sensitivity	Allihaibi et al., 2025 [41]	root level	root	independent clinical evaluation	Diagnocat	119	138	86.2	No	Table 2: 138 PARL-positive roots; 86.2%
Sensitivity	Boubaris et al., 2024 [45]		root	independent evaluation (500 roots)	Diagnocat	378	408	92.6	Yes	dichotomized from CBCTPAVI confusion matrix (Table 1): 408 lesion roots, 30 scored 0 by AI
Sensitivity	Calazans et al., 2022 [33]		image pair	internal hold-out (20% = 200 pairs)	Siamese DenseNet-121		109	64.5	Yes	Table 3 recall 0.6449 (complete dataset); positives approx. 20% of 546 lesion pairs
Sensitivity	Chau et al., 2025 [46]		lesion (scan)	internal 5-fold CV (185 scans)	CBCT-SAM	183	185	98.9	Yes	Table 7: 183/185 lesions detected
Sensitivity	Chen et al., 2024 [47]		root	internal hold-out (25 volumes)	Swin-UNETR-PRETRAIN-103	42	42	100.0	Yes	Table 1: sensitivity 1.00; 42 lesion roots in test set
Sensitivity	Ezhov et al., 2021 [48]		tooth	internal hold-out (300 scans)	Diagnocat		300	83.8	Yes	Table 3 periapical lesion 0.8383; test set >=300 positives per condition (minimum used)
Sensitivity	Fu et al., 2024 [50]	independent test set	tooth	internal independent test set (324 teeth, 36 AP)	PAL-Net	35	36	97.2	Yes	Table 2: sensitivity 0.97 of 36 AP teeth
Sensitivity	Fu et al., 2024 [50]	external 1	tooth	external (XSH; 393 teeth, 50 AP)	PAL-Net	47	50	94.0	No	0.94 of 50
Sensitivity	Fu et al., 2024 [50]	external 2	tooth	external (TSH; 464 teeth, 39 AP)	PAL-Net	35	39	89.7	No	0.90 of 39
Sensitivity	Fu et al., 2024 [50]	external 3	tooth	external (ZUSS; 929 teeth, 57 AP)	PAL-Net	50	57	87.7	No	0.88 of 57
Sensitivity	Hadzic et al., 2024 [42]		tooth	external validation (new clinical dataset)	CNN (Kirnbauer et al.)	260	300	86.7	Yes	Table 5: 260/300 lesion teeth
Sensitivity	Huang et al., 2024 [51]	BALD	lesion (root)	internal hold-out (20 slices)	Bayesian U-Net + BALD	21	25	84.0	Yes	Table 1: 84.0% of 25 lesions
Sensitivity	Huang et al., 2024 [51]	ME	lesion (root)	internal hold-out (20 slices)	Bayesian U-Net + max-entropy	19	25	76.0	No	76.0% of 25 lesions
Sensitivity	Huang et al., 2024 [51]	Bayesian U-Net	lesion (root)	internal hold-out (20 slices)	Bayesian U-Net (no AL)	8	25	32.0	No	32.0% of 25 lesions
Sensitivity	Lee et al., 2025 [52]		root	internal hold-out (30 volumes)	OAK-SSL		30	93.3	Yes	Table III per-root recall 0.933; number of lesion roots not reported (30 test volumes used)
Sensitivity	Naidu et al., 2025 [36]	ML	scan	internal hold-out (100 scans)	ML classifier	96	96	100.0	Yes	100% in 96 scans; positives/negatives not reported
Sensitivity	Naidu et al., 2025 [36]	DL	scan	internal hold-out (100 scans)	DL classifier		100	91.3	No	91.3%; denominator not reported, test set 100 scans used
Sensitivity	Orhan et al., 2020 [53]		lesion	independent evaluation (153 lesions)	Diagnocat	142	153	92.8	Yes	142/153 lesions detected (92.8%); authors also report recall 0.89
Sensitivity	Setzer et al., 2020 [54]		root	internal 5-fold CV (61 roots)	U-Net	27	29	93.1	Yes	27 TP / 29 lesion roots (text)
Specificity	Akalin and Özkan, 2025 [32]		image slice	internal hold-out (20%)	VGG16 + regularization 13	34	54	63.0	Yes	TN 34, FP 20
Specificity	Allihaibi et al., 2025 [41]	tooth level	tooth	independent clinical evaluation	Diagnocat	45	69	65.2	Yes	69 PARL-negative teeth; 65.2%
Specificity	Allihaibi et al., 2025 [41]	root level	root	independent clinical evaluation	Diagnocat	151	189	79.9	No	189 PARL-negative roots; 79.9%
Specificity	Boubaris et al., 2024 [45]		root	independent evaluation (500 roots)	Diagnocat	89	92	96.7	Yes	89/92 lesion-free roots scored 0 by AI
Specificity	Calazans et al., 2022 [33]		image pair	internal hold-out (20% = 200 pairs)	Siamese DenseNet-121		91	76.3	Yes	Table 3 specificity 0.7634; negatives approx. 20% of 454 healthy pairs

Metric	Study	Estimate	Unit	Test set	Model	Events	N	Value	Primary	Source and derivation
Specificity	Chen et al., 2024 [47]		root	internal hold-out (25 volumes)	Swin-UNETR-PRETRAIN-103	129	140	92.1	Yes	Table 1: specificity 0.92; 140 lesion-free roots (129 = 0.92x140 rounded)
Specificity	Ezhov et al., 2021 [48]		tooth	internal hold-out (300 scans)	Diagnocat		300	99.5	Yes	Table 3 periapical lesion 0.9953; >=300 negatives (minimum used)
Specificity	Fu et al., 2024 [50]	independent test set	tooth	internal independent test set (324 teeth, 36 AP)	PAL-Net	277	288	96.2	Yes	reconstructed from sensitivity 0.97, PPV 0.76 and 288 normal teeth (FP = 11)
Specificity	Fu et al., 2024 [50]	external 1	tooth	external (XSH)	PAL-Net	339	343	98.8	No	reconstructed (PPV 0.92; FP = 4)
Specificity	Fu et al., 2024 [50]	external 2	tooth	external (TSH)	PAL-Net	421	425	99.1	No	reconstructed (PPV 0.90; FP = 4)
Specificity	Fu et al., 2024 [50]	external 3	tooth	external (ZUSS)	PAL-Net	853	872	97.8	No	reconstructed (PPV 0.72; FP = 19)
Specificity	Hadzic et al., 2024 [42]		tooth	external validation (new clinical dataset)	CNN (Kirnbauer et al.)	2230	2647	84.2	Yes	Table 5: 2230/2647 lesion-free teeth
Specificity	Huang et al., 2024 [51]	BALD	root	internal hold-out (20 slices)	Bayesian U-Net + BALD	28	31	90.3	Yes	Table 1: 90.3% of 31 lesion-free roots (56 roots - 25 lesions)
Specificity	Huang et al., 2024 [51]	ME	root	internal hold-out (20 slices)	Bayesian U-Net + max-entropy	30	31	96.8	No	96.8% of 31
Specificity	Huang et al., 2024 [51]	Bayesian U-Net	root	internal hold-out (20 slices)	Bayesian U-Net (no AL)	30	31	96.8	No	96.8% of 31
Specificity	Naidu et al., 2025 [36]	ML	scan	internal hold-out (100 scans)	ML classifier	96	96	100.0	Yes	100% in 96 scans
Specificity	Naidu et al., 2025 [36]	DL	scan	internal hold-out (100 scans)	DL classifier		100	63.4	No	63.4%; denominator not reported
Specificity	Setzer et al., 2020 [54]		root	internal 5-fold CV (61 roots)	U-Net	28	32	87.5	Yes	28 TN / 32 lesion-free roots
Accuracy	Akalin and Özkan, 2025 [32]		image slice	internal hold-out (20%)	VGG16 + regularization 13	234	399	58.6	Yes	58.65%
Accuracy	Allihaibi et al., 2025 [41]	tooth level	tooth	independent clinical evaluation	Diagnocat	106	134	79.1	Yes	79.1% of 134 teeth
Accuracy	Allihaibi et al., 2025 [41]	root level	root	independent clinical evaluation	Diagnocat	270	327	82.6	No	82.6% of 327 roots
Accuracy	Boubaris et al., 2024 [45]		root	independent evaluation (500 roots)	Diagnocat	467	500	93.4	Yes	binary lesion presence: (378+89)/500
Accuracy	Calazans et al., 2022 [33]		image pair	internal hold-out (20% = 200 pairs)	Siamese DenseNet-121		200	70.0	Yes	Table 3 accuracy 0.70
Accuracy	Chen et al., 2024 [47]		root	internal hold-out (25 volumes)	Swin-UNETR-PRETRAIN-103	171	182	94.0	Yes	computed from reconstructed 2x2 (42+129)/182
Accuracy	Hadzic et al., 2024 [42]		tooth	external validation (new clinical dataset)	CNN (Kirnbauer et al.)	2490	2947	84.5	Yes	computed from published confusion matrix (Table 5)
Accuracy	Huang et al., 2024 [51]	BALD	root	internal hold-out (20 slices)	Bayesian U-Net + BALD	49	56	87.5	Yes	computed from 2x2: (21+28)/56
Accuracy	Naidu et al., 2025 [36]	ML	scan	internal hold-out (100 scans)	ML classifier	96	96	100.0	Yes	100% in 96 scans
Accuracy	Naidu et al., 2025 [36]	DL	scan	internal hold-out (100 scans)	DL classifier		100	84.6	No	84.62%
Accuracy	Setzer et al., 2020 [54]		root	internal 5-fold CV (61 roots)	U-Net	55	61	90.2	Yes	(27+28)/61 = 0.90; the 0.93 quoted in the abstract is the sensitivity
Accuracy	Zhao et al., 2024 [55]		image	internal hold-out (60 test images)	PAINet	56	60	93.3	Yes	4-class accuracy 0.9333 (56/60 original test images; metrics may have been computed on 480 augmented copies)
F1-score	Akalin and Özkan, 2025 [32]		image slice	internal hold-out (20%)	VGG16 + regularization 13		399	70.8	Yes	F1 70.80%
F1-score	Allihaibi et al., 2025 [41]	tooth level	tooth	independent clinical evaluation	Diagnocat		134	81.3	Yes	F1 81.3%; N = 134 teeth
F1-score	Allihaibi et al., 2025 [41]	root level	root	independent clinical evaluation	Diagnocat		327	80.7	No	F1 80.7%; N = 327 roots
F1-score	Arı et al., 2025 [44]		image	NR (300 images total)	Mask R-CNN / ResNet-101		300	87.0	Yes	F1 0.87 for periapical lesion (abstract); test-set size not verifiable - full text unavailable

Metric	Study	Estimate	Unit	Test set	Model	Events	N	Value	Primary	Source and derivation
F1-score	Calazans et al., 2022 [33]		image pair	internal hold-out (20% = 200 pairs)	Siamese DenseNet-121		200	69.7	Yes	Table 3 F1 0.6970
F1-score	Fu et al., 2024 [50]	independent test set	tooth	internal independent test set	PAL-Net		324	85.0	Yes	F1 0.85
F1-score	Fu et al., 2024 [50]	external 1	tooth	external (XSH)	PAL-Net		393	93.0	No	F1 0.93
F1-score	Fu et al., 2024 [50]	external 2	tooth	external (TSH)	PAL-Net		464	90.0	No	F1 0.90
F1-score	Fu et al., 2024 [50]	external 3	tooth	external (ZUSS)	PAL-Net		929	79.0	No	F1 0.79
F1-score	Orhan et al., 2020 [53]		lesion	independent evaluation (153 lesions)	Diagnocat		153	93.0	Yes	F-measure 0.93
F1-score	Zhao et al., 2024 [55]		image	internal hold-out (60 test images)	PAINet		60	93.4	Yes	macro F1 0.9336
AUC	Allihaibi et al., 2025 [41]	tooth level	tooth	independent clinical evaluation	Diagnocat		134	0.760	Yes	AUC 0.76; N = 134 teeth
AUC	Allihaibi et al., 2025 [41]	root level	root	independent clinical evaluation	Diagnocat		327	0.790	No	AUC 0.79; N = 327 roots
AUC	Fu et al., 2024 [50]	independent test set	tooth	internal independent test set	PAL-Net		324	0.970	Yes	AUC 0.97
AUC	Fu et al., 2024 [50]	external 1	tooth	external (XSH)	PAL-Net		393	0.960	No	AUC 0.96
AUC	Fu et al., 2024 [50]	external 2	tooth	external (TSH)	PAL-Net		464	0.950	No	AUC 0.95
AUC	Fu et al., 2024 [50]	external 3	tooth	external (ZUSS)	PAL-Net		929	0.930	No	AUC 0.93
AUC	Zhao et al., 2024 [55]		image	internal hold-out (60 test images)	PAINet		60	0.997	Yes	macro-average AUC 0.9972
Lesion DSC	Chau et al., 2025 [46]		scan	internal 5-fold CV (185 scans)	CBCT-SAM		185	0.700	Yes	Table 7: DSC 0.70 +/- 0.19
Lesion DSC	Chen et al., 2024 [47]		volume	internal hold-out (25 volumes)	Swin-UNETR-PRETRAIN-103		25	0.851	Yes	Table 1: lesion DICE 0.8512; N = 25 test volumes
Lesion DSC	Fu et al., 2024 [50]	independent test set	lesion	internal independent test set (36 lesions)	PAL-Net		36	0.810	Yes	DSC 0.81 (0.73-0.88)
Lesion DSC	Fu et al., 2024 [50]	external 1	lesion	external (XSH; 50 lesions)	PAL-Net		50	0.800	No	DSC 0.80
Lesion DSC	Fu et al., 2024 [50]	external 2	lesion	external (TSH; 39 lesions)	PAL-Net		39	0.900	No	DSC 0.90
Lesion DSC	Fu et al., 2024 [50]	external 3	lesion	external (ZUSS; 57 lesions)	PAL-Net		57	0.800	No	DSC 0.80
Lesion DSC	Huang et al., 2024 [51]	BALD	slice	internal hold-out (20 slices)	Bayesian U-Net + BALD		20	0.505	Yes	lesion Dice 0.505 (text; 0.504 abstract); N = 20 test slices
Lesion DSC	Huang et al., 2024 [51]	ME	slice	internal hold-out (20 slices)	Bayesian U-Net + max-entropy		20	0.501	No	lesion Dice 0.501
Lesion DSC	Huang et al., 2024 [51]	Bayesian U-Net	slice	internal hold-out (20 slices)	Bayesian U-Net (no AL)		20	0.288	No	lesion Dice 0.288
Lesion DSC	Lee et al., 2025 [52]		volume	internal hold-out (30 volumes)	OAK-SSL		30	0.647	Yes	Table I lesion Dice 0.647
Lesion DSC	Setzer et al., 2020 [54]		volume	internal 5-fold CV (20 volumes)	U-Net		20	0.670	Yes	DICE for actual true lesions 0.67 (overall lesion DICE incl. false negatives 0.52); N = 20 volumes

Value: percentage, except DSC and AUC (proportions). Events are blank where only a proportion was reported; N is then the test-set size used to approximate the variance. For estimates of 0% or 100%, 0.5 was added to events and non-events in the analysis. Primary, estimate designated as primary under the rules in Section 2.5.

(b) 2×2 tables used in the bivariate models.

Study	TP	FN	TN	FP	Use	Source and derivation
Akalin and Özkan, 2025 [32]	200	145	34	20	Primary bivariate model (exact)	Table 3 (Reg13): TP 200, FN 145 | TN 34, FP 20
Allihaibi et al., 2025 [41]	61	4	45	24	Primary bivariate model (exact)	Table 1/Suppl: 65 PARL-positive teeth; 93.8% | 69 PARL-negative teeth; 65.2%
Boubaris et al., 2024 [45]	378	30	89	3	Primary bivariate model (exact)	dichotomized from CBCTPAVI confusion matrix (Table 1): 408 lesion roots, 30 scored 0 by AI | 89/92 lesion-free roots scored 0 by AI
Chen et al., 2024 [47]	42	0	129	11	Primary bivariate model (exact)	Table 1: sensitivity 1.00; 42 lesion roots in test set | Table 1: specificity 0.92; 140 lesion-free roots (129 = 0.92x140 rounded)
Hadzic et al., 2024 [42]	260	40	2230	417	Primary bivariate model (exact)	Table 5: 260/300 lesion teeth | Table 5: 2230/2647 lesion-free teeth
Huang et al., 2024 [51]	21	4	28	3	Primary bivariate model (exact)	Table 1: 84.0% of 25 lesions | Table 1: 90.3% of 31 lesion-free roots (56 roots - 25 lesions)
Setzer et al., 2020 [54]	27	2	28	4	Primary bivariate model (exact)	27 TP / 29 lesion roots (text) | 28 TN / 32 lesion-free roots
Calazans et al., 2022 [33]	70	39	69	22	Extended model (approximate)	Table 3 recall 0.6449 (complete dataset); positives approx. 20% of 546 lesion pairs | Table 3 specificity 0.7634; negatives approx. 20% of 454 healthy pairs
Ezhov et al., 2021 [48]	251	49	299	1	Extended model (approximate)	Table 3 periapical lesion 0.8383; test set >=300 positives per condition (minimum used) | Table 3 periapical lesion 0.9953; >=300 negatives (minimum used)
Fu et al., 2024 [50]	35	1	277	11	Extended model (approximate)	Table 2: sensitivity 0.97 of 36 AP teeth | reconstructed from sensitivity 0.97, PPV 0.76 and 288 normal teeth (FP = 11)
Naidu et al., 2025 [36]	96	0	96	0	Not used (lesion-only sample)	100% in 96 scans; positives/negatives not reported | 100% in 96 scans

Exact, complete 2×2 table reported or reconstructed exactly from published counts or confusion matrices (for Chen et al. [47], the 129 true negatives were reconstructed from the reported specificity of 0.92 among 140 lesion-free roots). Approximate, reconstructed from reported proportions with an assumed or derived denominator. FN, false negatives; FP, false positives; TN, true negatives; TP, true positives.

Text S1. Study-by-study summary of reported standalone AI performance (values verified against the full text of each article; configuration designated as the primary estimate in the meta-analyses is marked *).

Aboelmaaty et al., 2024 [43]. Commercial platform (eVol DXS) used to localize the posterior superior alveolar artery in 120 scans with posterior maxillary periapical lesions and 120 controls; artery identified in 100% of scans; mucosal thickening more frequent when the artery lay beneath the sinus membrane (62.5% vs 8.6%). Lesion detection was not evaluated; the study contributes to the narrative synthesis only.

Akalin and Özkan, 2025 [32]. VGG16 transfer learning with 18 regularization strategies on the public UFPE dataset (2D coronal and sagittal slices; 80/20 split). Final configuration (regularization 13)*: TP 200, FP 20, FN 145, TN 34; accuracy 58.65%, recall 57.97%, specificity 62.96%, precision 90.91%, F1-score 70.80%. Unregularized processed-image baseline: recall 72.0%, precision 40.9%, F1-score 52.2%.

Allihaibi et al., 2025 [41]. Diagnocat evaluated on preoperative scans of 134 non-root-filled molars (327 roots) against two blinded endodontists. Tooth level*: 65 PARL-positive and 69 PARL-negative teeth; sensitivity 93.8%, specificity 65.2%, accuracy 79.1%, PPV 71.8%, NPV 91.8%, F1-score 81.3%, AUC 0.76. Root level: 138 positive and 189 negative roots; 86.2%, 79.9%, 82.6%, 75.8%, 88.8%, 80.7%, AUC 0.79. Postoperative scans: PPV 54.2% (teeth) and 46.9% (roots); F1-score 67.2% and 59.2%.

Arı et al., 2025 [44]. Mask R-CNN (ResNet-101) trained to segment 15 conditions on 300 CBCT images; F1-score 0.87 for periapical lesion*. Only the abstract was available; the test-set size could not be verified.

Boubaris et al., 2024 [45]. Diagnocat versus semi-automated segmentation (Mimics) for CBCTPAVI scoring of 500 roots (408 with lesions, 92 root-filled without lesion). Exact score agreement 84.4%; no root scored 1 or 2 by the AI. Dichotomized lesion presence from the published confusion matrix*: 378/408 lesion roots detected (92.6%), 89/92 lesion-free roots scored 0 (96.7%), accuracy 93.4%.

Calazans et al., 2022 [33]. Siamese network with VGG-16 or DenseNet-121 backbones on 1000 pairs of sagittal and coronal sections (454 healthy, 276 small lesions, 270 large lesions; 60/20/20 split). Complete dataset, DenseNet-121*: accuracy 0.700, F1-score 0.697, specificity 0.763, precision 0.758, recall 0.645. Healthy versus large lesions: accuracy 0.79-0.81, specificity 0.91-0.92, recall 0.56-0.59.

Chau et al., 2025 [46]. CBCT-SAM (with progressive prediction refinement) versus CBCT-SAM without PPR, modified U-Net and PAL-Net; 185 lesion-positive scans; 5-fold cross-validation. CBCT-SAM*: 183/185 lesions detected (98.9%); voxel-level segmentation accuracy 99.65%, sensitivity 72.4%, specificity 99.9%, precision 0.73, DSC 0.70 ± 0.19.

Chen et al., 2024 [47]. U-Net and Swin-UNETR (pretrained or from scratch) trained with 20-103 volumes; 25 test volumes with 42 lesion roots and 140 lesion-free roots. Swin-UNETR-PRETRAIN-103*: lesion DICE 0.8512; per-root sensitivity 1.00, specificity 0.92. Swin-UNETR-PRETRAIN-60: DICE 0.8395, sensitivity 1.00, specificity 0.94. U-Net-103: DICE 0.8461, sensitivity 1.00, specificity 0.91.

Ezhov et al., 2021 [48]. Diagnocat trained on 1346 scans; test set of 300 scans sampled to contain at least 300 positive and 300 negative teeth per condition. Standalone periapical-lesion module*: sensitivity 0.8383, specificity 0.9953 (poorly circumscribed radiolucency 0.70/0.99; well-circumscribed 0.73/0.997). Cross-condition aggregate: 0.924/0.990. Reader study (24 dentists, 30 scans): aggregate sensitivity 0.854 aided versus 0.767 unaided.

Flores et al., 2009 [49]. Graph-based random-walk segmentation with LDA-AdaBoost classification of 17 lesions as cyst or granuloma; leave-one-out cross-validation; accuracy 94.1% against expert CBCT diagnosis and 88.2% against biopsy. Differential diagnosis; not pooled.

Fu et al., 2024 [50]. PAL-Net (3D CNN); internal 5-fold cross-validation (2841 teeth, 381 AP), independent internal test set (324 teeth, 36 AP)* and three external sets (393/464/929 teeth; 50/39/57 AP). Sensitivity 0.99 (CV), 0.97 (test), 0.94/0.90/0.88 (external); PPV 0.80, 0.76, 0.92/0.90/0.72; F1-score 0.88, 0.85, 0.93/0.90/0.79; AUC 0.98, 0.97, 0.96/0.95/0.93; DSC 0.87, 0.81, 0.80/0.90/0.80. Specificity was not reported and was reconstructed from sensitivity, PPV and prevalence (test set 0.962; external 0.988/0.991/0.978). Reader study: junior dentists' AUC 0.89→0.94, senior 0.91→0.93 with assistance; reading time reduced by 69.3 and 32.4 min per 100 scans.

Hadzic et al., 2024 [42]. Previously developed CNN evaluated on 195 clinically representative scans (2947 present teeth; 300 lesions by three-investigator consensus)*: TP 260, FN 40, FP 417, TN 2230; sensitivity 86.7%, specificity

84.2%; upper jaw 87.8%/82.3%, lower jaw 84.6%/86.4%; sensitivity 90.4% excluding PAI-1 lesions; 42 false positives at 669 missing-tooth sites.

Huang et al., 2024 [51]. Bayesian U-Net with BALD or maximum-entropy active learning versus no active learning; 20 test slices containing 25 lesions on 56 roots. BALD*: sensitivity 84.0% (21/25), specificity 90.3% (28/31), precision 87.5%, lesion Dice 0.505. ME: 76.0%, 96.8%, 95.0%, 0.501. Bayesian U-Net without AL: 32.0%, 96.8%, 88.9%, 0.288.

Lee et al., 2025 [52]. OAK-SSL semi-supervised model (20 labelled + 80 unlabelled training volumes; 30 test volumes with small lesions)*: lesion Dice 0.647 (UA-MT 0.588, supervised U-Net 0.215); per-root precision 0.791, recall 0.933; one anatomically implausible lesion prediction.

Naidu et al., 2025 [36]. ML and DL classification of periapical cysts, abscesses and granulomas; 400 training and 100 test scans, all lesion-positive. ML*: 100% sensitivity, specificity and accuracy in 96 scans. DL: sensitivity 91.3%, specificity 63.4%, accuracy 84.62%; denominators not reported.

Orhan et al., 2020 [53]. Diagnocat on 153 lesions in 109 patients identified by one radiologist*: 142/153 lesions detected (92.8%); reported recall 0.89, precision 0.95, F-measure 0.93; AI and manual lesion volumes not significantly different (Bland-Altman mean difference 0.15 mm).

Setzer et al., 2020 [54]. U-Net on 20 limited-FOV volumes (61 roots; 29 with lesions) with 5-fold cross-validation*: TP 27, FN 2, FP 4, TN 28; sensitivity 0.93, specificity 0.88, PPV 0.87, NPV 0.93, accuracy 0.90; lesion DICE 0.52 overall (range 0.36-0.70) and 0.67 for true lesions.

Zhao et al., 2024 [55]. PAINet versus ResNet, Swin Transformer-Large, VOLO-D1 and attention models for four-class CBCTPAI grading (normal, CBCTPAI 1-2, CBCTPAI 3-5, young permanent teeth); 15 original test images per class (480 after augmentation). PAINet*: accuracy 0.9333, precision 0.9415, recall 0.9333, F1-score 0.9336, macro-average AUC 0.9972, $\kappa = 0.911$.

Figure S1.

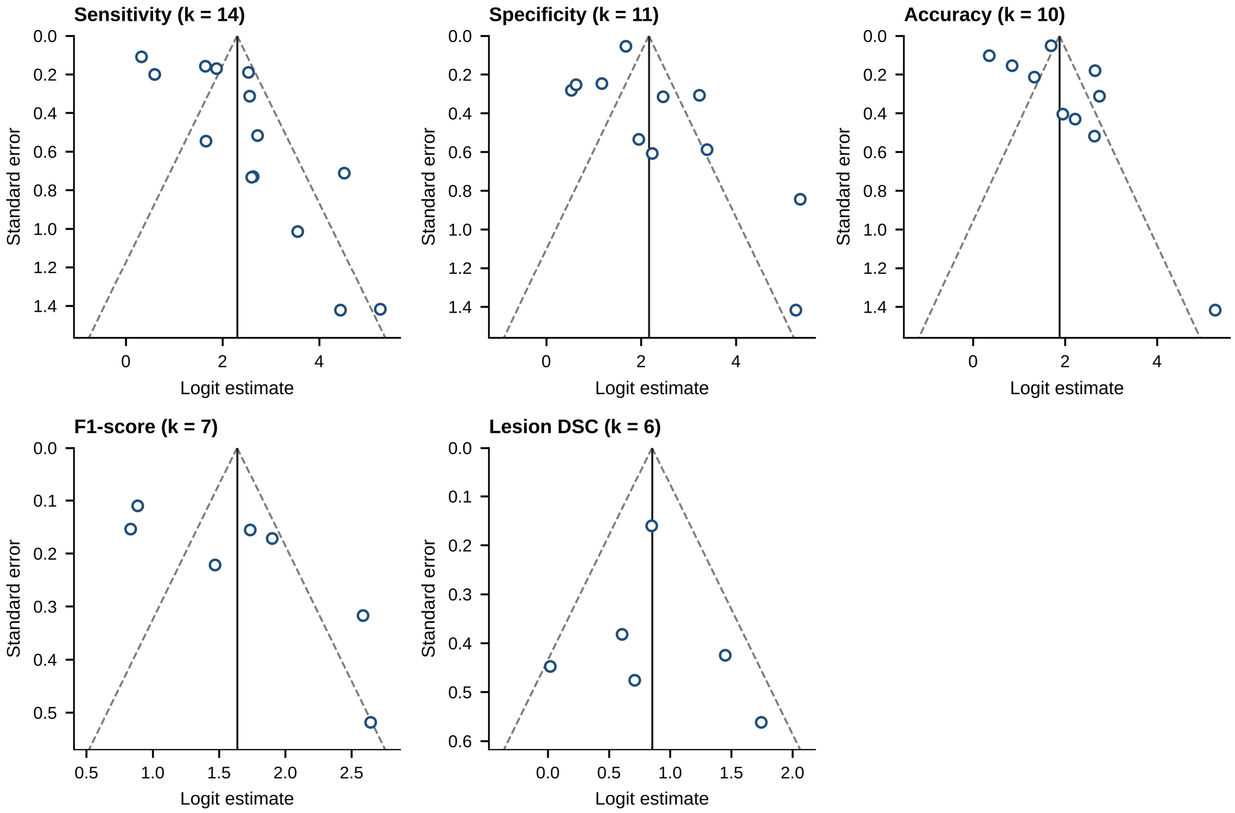

Figure S1. Funnel plots for sensitivity, specificity, accuracy, F1-score, and lesion Dice similarity coefficient (DSC), one estimate per study. Circles, study estimates on the logit scale; solid line, pooled estimate; dashed lines, pseudo-95% confidence limits. Presented for transparency only; because each analysis included few studies and funnel-plot asymmetry is a poor indicator of publication bias in diagnostic accuracy reviews, asymmetry was not interpreted.

Table S8. PRISMA 2020 checklist. Locations refer to sections, tables, and figures of the main manuscript unless prefixed with "S".

Section and topic	Item #	Checklist item	Location where item is reported
**TITLE**			
Title	1	Identify the report as a systematic review.	Title ("A Systematic Review and Meta-Analysis")
**ABSTRACT**			
Abstract	2	See the PRISMA 2020 for Abstracts checklist.	Abstract; Table S9
**INTRODUCTION**			
Rationale	3	Describe the rationale for the review in the context of existing knowledge.	Section 1, paragraphs 1-5
Objectives	4	Provide an explicit statement of the objective(s) or question(s) the review addresses.	Section 1, final paragraph
**METHODS**			
Eligibility criteria	5	Specify the inclusion and exclusion criteria for the review and how studies were grouped for the syntheses.	Section 2.2 (PIRD criteria, exclusions); Sections 2.5 and 2.8 (grouping by AI task and metric; eligibility of each metric)
Information sources	6	Specify all databases, registers, websites, organisations, reference lists and other sources searched or consulted to identify studies. Specify the date when each source was last searched or consulted.	Section 2.3; Table S1 (all sources last searched 12 December 2025)
Search strategy	7	Present the full search strategies for all databases, registers and websites, including any filters and limits used.	Section 2.3 (limits); Table S1 (full strategies)
Selection process	8	Specify the methods used to decide whether a study met the inclusion criteria of the review, including how many reviewers screened each record and each report retrieved, whether they worked independently, and if applicable, details of automation tools used in the process.	Section 2.4
Data collection process	9	Specify the methods used to collect data from reports, including how many reviewers collected data from each report, whether they worked independently, any processes for obtaining or confirming data from study investigators, and if applicable, details of automation tools used in the process.	Sections 2.5 and 2.6
Data items	10a	List and define all outcomes for which data were sought. Specify whether all results that were compatible with each outcome domain in each study were sought, and if not, the methods used to decide which results to collect.	Section 2.2 (outcomes); Section 2.5 (all reported metrics; rules for primary and secondary estimates)
	10b	List and define all other variables for which data were sought. Describe any assumptions made about any missing or unclear information.	Section 2.5 (variables, including input data and training-set size); Section 2.6 (missing information recorded as NR; no imputation)
Study risk of bias assessment	11	Specify the methods used to assess risk of bias in the included studies, including details of the tool(s) used, how many reviewers assessed each study and whether they worked independently, and if applicable, details of automation tools used in the process.	Section 2.7; Table 1
Effect measures	12	Specify for each outcome the effect measure(s) used in the synthesis or presentation of results.	Section 2.2 (outcomes); Section 2.9 (logit-transformed proportions; bivariate summary sensitivity and specificity)
Synthesis methods	13a	Describe the processes used to decide which studies were eligible for each synthesis.	Sections 2.5 (primary-estimate and metric-eligibility rules), 2.9 and 2.10
	13b	Describe any methods required to prepare the data for presentation or synthesis, such as handling of missing summary statistics, or data conversions.	Section 2.5 (denominators and their reconstruction); Section 2.9 (logit transformation; continuity correction); Table S7
	13c	Describe any methods used to tabulate or visually display results of individual studies and syntheses.	Section 2.8; Tables 2, 3 and 5; Figures 4-9; Table S3
	13d	Describe any methods used to synthesize results and provide a rationale for the choice(s). If meta-analysis was performed, describe the model(s), method(s) to identify the presence and extent of statistical heterogeneity, and software package(s) used.	Sections 2.8-2.10
	13e	Describe any methods used to explore possible causes of heterogeneity among study results.	Section 2.11; Tables S3 and S4
	13f	Describe any sensitivity analyses conducted to assess robustness of the synthesized results.	Sections 2.10 and 2.11 (one-estimate-per-study, single-level and three-level models; leave-one-study-out; restricted analyses)
Reporting bias assessment	14	Describe any methods used to assess risk of bias due to missing results in a synthesis (arising from reporting biases).	Section 2.11
Certainty assessment	15	Describe any methods used to assess certainty (or confidence) in the body of evidence for an outcome.	Section 2.12
**RESULTS**			

Section and topic	Item #	Checklist item	Location where item is reported
Study selection	16a	Describe the results of the search and selection process, from the number of records identified in the search to the number of studies included in the review, ideally using a flow diagram.	Section 3.1; Figure 1
	16b	Cite studies that might appear to meet the inclusion criteria, but which were excluded, and explain why they were excluded.	Section 3.1; Table S2
Study characteristics	17	Cite each included study and present its characteristics.	Section 3.2; Tables 2 and 3; Tables S3 and S4
Risk of bias in studies	18	Present assessments of risk of bias for each included study.	Section 3.3; Table 4; Figures 2 and 3
Results of individual studies	19	For all outcomes, present, for each study: (a) summary statistics for each group (where appropriate) and (b) an effect estimate and its precision (e.g., confidence/credible interval), ideally using structured tables or plots.	Section 3.4; Table 3; Figures 5-9; Table S7; Text S1
Results of syntheses	20a	For each synthesis, briefly summarise the characteristics and risk of bias among contributing studies.	Section 3.5; Table 5 (footnotes); Table S3
	20b	Present results of all statistical syntheses conducted. If meta-analysis was done, present for each the summary estimate and its precision (e.g., confidence/credible interval) and measures of statistical heterogeneity. If comparing groups, describe the direction of the effect.	Sections 3.5.1-3.5.3; Table 5; Figures 4-9
	20c	Present results of all investigations of possible causes of heterogeneity among study results.	Section 3.5.4
	20d	Present results of all sensitivity analyses conducted to assess the robustness of the synthesized results.	Sections 3.5.1-3.5.4; Tables S5 and S6
Reporting biases	21	Present assessments of risk of bias due to missing results (arising from reporting biases) for each synthesis assessed.	Section 2.11; Figure S1; Table 6 (footnote e)
Certainty of evidence	22	Present assessments of certainty (or confidence) in the body of evidence for each outcome assessed.	Section 3.6; Table 6
**DISCUSSION**			
Discussion	23a	Provide a general interpretation of the results in the context of other evidence.	Sections 4.1-4.4
	23b	Discuss any limitations of the evidence included in the review.	Section 4.5, second paragraph
	23c	Discuss any limitations of the review processes used.	Section 4.5, third paragraph
	23d	Discuss implications of the results for practice, policy, and future research.	Section 4.6; Section 5
**OTHER INFORMATION**			
Registration and protocol	24a	Provide registration information for the review, including register name and registration number, or state that the review was not registered.	Section 2.1; Abstract (PROSPERO CRD420251184440)
	24b	Indicate where the review protocol can be accessed, or state that a protocol was not prepared.	Section 2.1 (PROSPERO record URL)
	24c	Describe and explain any amendments to information provided at registration or in the protocol.	Section 2.1 (deviations from the registered protocol)
Support	25	Describe sources of financial or non-financial support for the review, and the role of the funders or sponsors in the review.	Funding; Acknowledgments; Conflicts of Interest (role of funder)
Competing interests	26	Declare any competing interests of review authors.	Conflicts of Interest
Availability of data, code and other materials	27	Report which of the following are publicly available and where they can be found: template data collection forms; data extracted from included studies; data used for all analyses; analytic code; any other materials used in the review.	Data Availability Statement; Tables S3 and S7; analysis code (Supplementary Materials)

From: Page, M.J.; McKenzie, J.E.; Bossuyt, P.M.; et al. The PRISMA 2020 statement: an updated guideline for reporting systematic reviews. BMJ 2021, 372, n71. https://doi.org/10.1136/bmj.n71. NR, not reported; PIRD, Population, Index test, Reference standard, Diagnosis of interest.

Table S9. PRISMA 2020 for Abstracts checklist.

Section and topic	Item #	Checklist item	Reported
**TITLE**			
Title	1	Identify the report as a systematic review.	Yes (title)
**BACKGROUND**			
Objectives	2	Provide an explicit statement of the main objective(s) or question(s) the review addresses.	Yes (Background/Objectives)
**METHODS**			
Eligibility criteria	3	Specify the inclusion and exclusion criteria for the review.	Yes (Methods)
Information sources	4	Specify the information sources (e.g., databases, registers) used to identify studies and the date when each was last searched.	Yes (Methods)
Risk of bias	5	Specify the methods used to assess risk of bias in the included studies.	Yes (Methods: QUADAS-2)
Synthesis of results	6	Specify the methods used to present and synthesise results.	Yes (Methods)
**RESULTS**			
Included studies	7	Give the total number of included studies and participants and summarise relevant characteristics of studies.	Yes (Results)
Synthesis of results	8	Present results for main outcomes, preferably indicating the number of included studies and participants for each. If meta-analysis was done, report the summary estimate and confidence/credible interval. If comparing groups, indicate the direction of the effect.	Yes (Results)
**DISCUSSION**			
Limitations of evidence	9	Provide a brief summary of the limitations of the evidence included in the review (e.g., study risk of bias, inconsistency and imprecision).	Yes (Results: heterogeneity, GRADE; Conclusions)
Interpretation	10	Provide a general interpretation of the results and important implications.	Yes (Conclusions)
**OTHER**			
Funding	11	Specify the primary source of funding for the review.	Reported in the Funding statement; the journal's structured abstract format does not include a funding field
Registration	12	Provide the register name and registration number.	Yes (Methods: PROSPERO CRD420251184440)

From: Page, M.J.; et al. BMJ 2021, 372, n71.

Table S10. PRISMA-DTA checklist. Locations refer to sections, tables, and figures of the main manuscript unless prefixed with “S”. Items numbered D1 and D2 are specific to diagnostic test accuracy reviews; item numbers 15 and 23 of the original PRISMA checklist are not used in PRISMA-DTA.

Section and topic	Item #	Checklist item	Location where item is reported
**TITLE / ABSTRACT**			
Title	1	Identify the report as a systematic review (± meta-analysis) of diagnostic test accuracy (DTA) studies.	Title
Abstract	2	Abstract: See PRISMA-DTA for abstracts.	Abstract; Table S11
**INTRODUCTION**			
Rationale	3	Describe the rationale for the review in the context of what is already known.	Section 1, paragraphs 1-5
Clinical role of index test	D1	State the scientific and clinical background, including the intended use and clinical role of the index test, and if applicable, the rationale for minimally acceptable test accuracy (or minimum difference in accuracy for comparative design).	Section 1, paragraphs 3-5 (CBCT interpretation burden; AI as computer-assisted interpretation); Section 4.6 (intended use as decision support). A minimally acceptable accuracy was not prespecified.
Objectives	4	Provide an explicit statement of question(s) being addressed in terms of participants, index test(s), and target condition(s).	Section 1, final paragraph; Section 2.2
**METHODS**			
Protocol and registration	5	Indicate where the review protocol can be accessed (e.g., web address), and, if available, provide registration information including registration number.	Section 2.1 (PROSPERO CRD420251184440; protocol URL)
Eligibility criteria	6	Specify study characteristics (participants, setting, index test(s), reference standard(s), target condition(s), and study design) and report characteristics (e.g., years considered, language, publication status) used as criteria for eligibility, giving rationale.	Section 2.2 (PIRD criteria; no restriction on setting or language; exclusions)
Information sources	7	Describe all information sources (e.g., databases with dates of coverage, contact with authors to identify additional studies) in the search and date last searched.	Section 2.3; Table S1 (all sources last searched 12 December 2025)
Search	8	Present full search strategies for all electronic databases and other sources searched, including any limits used, such that they could be reproduced.	Section 2.3 (limits); Table S1
Study selection	9	State the process for selecting studies (i.e., screening, eligibility, included in systematic review, and, if applicable, included in the meta-analysis).	Section 2.4; Sections 2.5 and 2.9 (eligibility for each meta-analysis)
Data collection process	10	Describe method of data extraction from reports (e.g., piloted forms, independently, in duplicate) and any processes for obtaining and confirming data from investigators.	Sections 2.5 and 2.6
Definitions for data extraction	11	Provide definitions used in data extraction and classifications of target condition(s), index test(s), reference standard(s) and other characteristics (e.g., study design, clinical setting).	Section 1, paragraph 2 (target condition); Section 2.2; Section 2.5 (extracted items and metric definitions); Table S4
Risk of bias and applicability	12	Describe methods used for assessing risk of bias in individual studies and concerns regarding the applicability to the review question.	Section 2.7; Table 1
Diagnostic accuracy measures	13	State the principal diagnostic accuracy measure(s) reported (e.g., sensitivity, specificity) and state the unit of assessment (e.g., per-patient, per-lesion).	Section 2.2 (outcomes); Section 2.5 (unit of analysis; tooth level primary); Sections 2.9-2.10
Synthesis of results	14	Describe methods of handling data, combining results of studies and describing variability between studies. This could include, but is not limited to: (a) handling of multiple definitions of target condition; (b) handling of multiple thresholds of test positivity; (c) handling multiple index test readers; (d) handling of indeterminate test results; (e) grouping and comparing tests; (f) handling of different reference standards.	Sections 2.5 and 2.8-2.11. (a) Section 2.5 (lesion detection vs. lesion-type classification; dichotomization of grading); (b) each study reported a single operating point, so threshold effects were examined only through the bivariate correlation (Section 2.9); (c) standalone AI only, AI-assisted readers summarized narratively (Section 2.5); (d) indeterminate results were not reported by the included studies; (e) grouping by AI task (Section 2.8) and descriptive comparison of independent evaluations vs. development studies (Section 2.11); (f) reference standards tabulated and explored descriptively (Section 2.11; Table S4)
Meta-analysis	D2	Report the statistical methods used for meta-analyses, if performed.	Sections 2.9 and 2.10
Additional analyses	16	Describe methods of additional analyses (e.g., sensitivity or subgroup analyses, meta-regression), if done, indicating which were pre-specified.	Sections 2.10 and 2.11; analyses added after registration are listed as deviations in Section 2.1
**RESULTS**			
Study selection	17	Provide numbers of studies screened, assessed for eligibility, included in the review (and included in meta-analysis, if applicable) with reasons for exclusions at each stage, ideally with a flow diagram.	Section 3.1; Figure 1; Table S2; Table 5 (studies in each meta-analysis)

Section and topic	Item #	Checklist item	Location where item is reported
Study characteristics	18	For each included study provide citations and present key characteristics including: (a) participant characteristics (presentation, prior testing); (b) clinical setting; (c) study design; (d) target condition definition; (e) index test; (f) reference standard; (g) sample size; (h) funding sources.	Section 3.2; Tables 2 and 3; Tables S3 and S4. (h) Funding sources of the included studies were not extracted.
Risk of bias and applicability	19	Present evaluation of risk of bias and concerns regarding applicability for each study.	Section 3.3; Table 4; Figures 2 and 3
Results of individual studies	20	For each analysis in each study (e.g., unique combination of index test, reference standard, and positivity threshold) report 2×2 data (TP, FP, FN, TN), estimates of diagnostic accuracy and confidence intervals, ideally with a forest or receiver operator characteristic (ROC) plot.	Table 3; Figures 4-9; Table S7 (per-estimate data) and Table S7b (2×2 data); Text S1
Synthesis of results	21	Describe test accuracy, including variability; if meta-analysis was done, include results and confidence intervals.	Sections 3.5.1-3.5.3; Table 5; Figures 4-9
Additional analysis	22	Give results of additional analyses, if done (e.g., sensitivity or subgroup analyses, meta-regression; analysis of index test: failure rates, proportion of inconclusive results, adverse events).	Section 3.5.4; Tables S5 and S6. Failure rates and inconclusive results were not reported by the included studies.
**DISCUSSION**			
Summary	24	Summarize the main findings including the strength of evidence.	Section 4.1; Section 3.6 and Table 6 (GRADE)
Limitations	25	Discuss limitations from included studies (e.g., risk of bias and concerns regarding applicability) and from the review process (e.g., incomplete retrieval of identified research).	Section 4.5, paragraphs 2 and 3
Conclusions	26	Provide a general interpretation of the results in the context of other evidence. Discuss implications for future research and clinical practice (e.g., the intended use and clinical role of the index test).	Sections 4.2-4.4 and 4.6; Section 5
**OTHER**			
Funding	27	For the systematic review, describe the sources of funding and other support and the role of the funders.	Funding; Acknowledgments; Conflicts of Interest (role of funder)

From: McInnes, M.D.F.; Moher, D.; Thombs, B.D.; et al. Preferred Reporting Items for a Systematic Review and Meta-analysis of Diagnostic Test Accuracy Studies: The PRISMA-DTA Statement. JAMA 2018, 319, 388-396. https://doi.org/10.1001/jama.2017.19163. AI, artificial intelligence; CBCT, cone-beam computed tomography; GRADE, Grading of Recommendations Assessment, Development and Evaluation.

Table S11. PRISMA-DTA for Abstracts checklist.

Section and topic	Item #	Checklist item	Reported
**TITLE AND PURPOSE**			
Title	1	Identify the report as a systematic review (± meta-analysis) of diagnostic test accuracy (DTA) studies.	Yes (title)
Objectives	2	Indicate the research question, including components such as participants, index test, and target conditions.	Yes (Background/Objectives)
**METHODS**			
Eligibility criteria	3	Include study characteristics used as criteria for eligibility.	Yes (Methods)
Information sources	4	List the key databases searched and the search dates.	Yes (Methods)
Risk of bias and applicability	5	Indicate the methods of assessing risk of bias and applicability.	Yes (Methods: QUADAS-2)
Synthesis of results	A1	Indicate the methods for the data synthesis.	Yes (Methods)
**RESULTS**			
Included studies	6	Indicate the number and type of included studies and the participants and relevant characteristics of the studies (including the reference standard).	Yes (Results: 18 studies; detection and segmentation counts; retrospective, internally validated)
Synthesis of results	7	Include the results for the analysis of diagnostic accuracy, preferably indicating the number of studies and participants. Describe test accuracy including variability; if meta-analysis was done, include summary results and confidence intervals.	Yes (Results)
**DISCUSSION**			
Strengths and limitations	9	Provide a brief summary of the strengths and limitations of the evidence.	Yes (Results: heterogeneity, prediction intervals, GRADE; Conclusions)
Interpretation	10	Provide a general interpretation of the results and the important implications.	Yes (Conclusions)
**OTHER**			
Funding	11	Indicate the primary source of funding for the review.	Reported in the Funding statement; the journal's structured abstract format has no funding field
Registration	12	Provide the registration number and the registry name.	Yes (Methods: PROSPERO CRD420251184440)

From: Cohen, J.F.; Deeks, J.J.; Hooft, L.; et al. Preferred reporting items for journal and conference abstracts of systematic reviews and meta-analyses of diagnostic test accuracy studies (PRISMA-DTA for Abstracts): checklist, explanation, and elaboration. BMJ 2021, 372, n265. https://doi.org/10.1136/bmj.n265. Item number 8 is not used in PRISMA-DTA for Abstracts.
